# Activated Neutrophils Secrete Chitinase-Like 1 and Attenuate Liver Inflammation by Inhibiting Pro-Inflammatory Macrophage Responses

**DOI:** 10.3389/fimmu.2022.824385

**Published:** 2022-04-21

**Authors:** Yu Lu, Na Chang, Xinhao Zhao, Renmin Xue, Jing Liu, Lin Yang, Liying Li

**Affiliations:** Department of Cell Biology, Municipal Laboratory for Liver Protection and Regulation of Regeneration, Capital Medical University, Beijing, China

**Keywords:** activated neutrophil, liver inflammation, Chitinase-like 1, pro-inflammatory macrophage, Sphingosine 1-phosphate

## Abstract

Excessive activation and recruitment of neutrophils are generally considered to be associated with pathological aggravation of multiple diseases. However, as the role of neutrophils in tissue injury repair is receiving increasing attention, it is necessary to further explore the beneficial role of activated neutrophils in promoting the resolution of inflammation after injury. In this study, we found that activated neutrophils have a crucial function in suppressing liver inflammation. In methionine-choline-deficient and high-fat (MCDHF) diet induced liver inflammation in mice, tail vein injection of activated neutrophils (A-Neu, stimulated by sphingosine 1-phosphate) inhibited the expressions of pro-inflammatory cytokines in the liver, including C-C chemokine motif ligand 4, tumor necrosis factor and nitric oxide synthase 2, and attenuated liver injury. However, non-activated neutrophils (N-Neu) did not have these effects. *In vitro*, pro-inflammatory macrophages were co-cultured with N-Neu or A-Neu by transwell, respectively. A-Neu was found to suppress the pro-inflammatory phenotype of macrophages by using RT-qPCR, western blot and cytometric bead array. Microarray analysis showed that there were systematic variations in transcript expression levels between N-Neu and A-Neu. GeneVenn software was used to show the gene expression overlap between GO terms including Regulation of Cell Communication, Cytokine Secretion, Inflammatory Response and Extracellular Space clusters. We identified that Chitinase-like 1 (CHIL1) secreted by S1P activated neutrophils may be an important mediators affecting the pro-inflammatory macrophage responses. In the injured liver of mice induced by MCDHF diet, the expression of *Chil1* mRNA increased and was positively correlated with the neutrophil marker *Ly6g*. Moreover, the secretion of CHIL1 in A-Neu increased significantly. Strikingly, the effect of A-Neu on macrophage response was reproduced by incubating pro-inflammatory macrophages with recombinant CHIL1. A-Neu conditioned medium were incubated with CHIL1 antibody-conjugated protein G beads, magnetically separated to immunodepletion CHIL1 from the A-Neu supernatant, which can partially weaken its inhibitory effect of A-Neu on the production of macrophage pro-inflammatory cytokines. Together, the conclusions indicated that A-Neu could inhibit the pro-inflammatory macrophage responses by secreting CHIL1, thereby effectively inhibiting liver inflammation.

## Introduction

Neutrophils are the vanguard of inflammatory monocytes and are the first cells to be recruited to the injured inflammatory site (1, 2). They are traditionally been thought that their role is limited to eliminating pathogens in the process of innate immune response by producing neutrophil extracellular traps (NET), releasing cytotoxic granular contents, producing reactive oxygen species (ROS), and many other mechanisms (3, 4). However, these powerful functions of neutrophils often lead to more uncontrolled inflammation and exacerbated tissue damage (2, 5, 6). Therefore, neutrophils are usually considered harmful cells under various inappropriate inflammatory conditions. To date, the therapeutic strategy for inflammatory diseases has been to inhibit the recruitment of neutrophils to allow repair. However, this simplistic view may be flawed, because neutrophils have recently been considered to be the key to tissue repair after injury.

Neutrophils have recently been thought to contribute to host protection in a variety of environments. Depletion of neutrophils will lead to impaired hematopoietic recovery after genotoxic injury and complete loss of endothelial cell proliferation, thus impairing early angiogenesis (7). In addition, the maturation and function of natural killer cell will also be impaired (8). In a fully repairing sterile thermal hepatic injury, neutrophils penetrate the injury site, and perform the critical task of removing damaged blood vessels and creating new vascular (9). In acid induced lung injury, the accumulation of DNA fragments in the lung increases, and neutrophils can phagocytize and degrade extracellular DNA fragments in a MyD88 dependent manner to achieve organ repair (10). In myocardial infarction, activated neutrophils secrete neutrophil gelatinase-associated lipoprotein, which polarizes macrophages toward the M2c phenotype, whose primary role is to clear apoptotic cells, thereby promoting cardiac healing and prognosis (11). In a mouse model of liver inflammation regression, neutrophils mediate silencing of pro-inflammatory macrophage NLRP3 inflammasome in the liver *via* mir-223, inducing their polarization toward a restorative phenotype, thereby resolving inflammation and early fibrosis (12). A large number of published studies have demonstrated the critical role of neutrophils in tissue repair after injury. However, the mechanisms by which neutrophils promote the resolution of inflammation remain unclear.

Within hours after neutrophils arrive at the site of inflammation, macrophages are also recruited to the damaged tissue (13). Macrophages have phenotypic plasticity. Traditionally, activated macrophages are divided into pro-inflammatory macrophages that mediate tissue injury and inflammation, and reparative macrophages that promote tissue repair (14, 15). Although this simple classification does not fully reflect the heterogeneity of macrophages. Pro-inflammatory macrophages are characterized by the high expressions of pro-inflammatory cytokines: C-C chemokine motif ligand 4 (CCL4), tumor necrosis factor (TNF) and nitric oxide synthase 2 (NOS2), etc, while reparative macrophages are characterized by high expressions of Arginase 1, CD163 and CD206 (16, 17). Under the control of endogenous signal mediators, pro-inflammatory macrophages are reprogrammed into reparative macrophages to promote wound healing and restore tissue homeostasis (18). Phenotypic plasticity of macrophages is essential for effective repair, and local tissue signals have a considerable impact on the phenotypic transformation of macrophages. However, the role of neutrophils in macrophage phenotypic reprogramming needs further investigation. It has been shown that NETs released by activated neutrophils can polarize macrophages toward the M2b phenotype, which is involved in the suppression and regulation of inflammatory responses (19). It was also recently reported that activated neutrophils from acetaminophen (APAP) challenged mice livers promote the phenotypic transformation from pro-inflammatory macrophages to reparative macrophages by releasing ROS, while non-activated neutrophils from bone marrow do not have this effect (20). In this context, further research is needed to better understand the difference between activated neutrophils and non-activated neutrophils on the phenotypic transformation of macrophages.

Liver inflammation is a major factor in liver pathology during chronic injury of different etiologies (21, 22). Here, we used the methionine-choline-deficient and high-fat (MCDHF) diet induced liver injury model to study the role of neutrophils in liver inflammation. We found that activated neutrophils (A-Neu) could inhibit the expressions of pro-inflammatory cytokines *in vivo* and *in vitro* and ameliorate MCDHF-induced liver injury in mice, but non-activated neutrophils (N-Neu) did not. Mechanistically, A-Neu inhibited the pro-inflammatory responses of macrophage by secreting Chitinase-like 1 (CHIL1) for optimal liver repair. Our data demonstrated the beneficial role of A-Neu in liver inflammation and provided new ideas for the treatment of inflammatory liver diseases.

## Materials and Methods

### Reagents

RPMI 1640 was from Invitrogen (Grand Island, NY). Fetal bovine serum was from HyClone/Thermo Scientific (Logan, UT). PCR reagents were from Applied Biosystems (Foster City, CA). Sphingosine 1-phosphate (S1P, Cay62570-1) was from Biomol (Tebu, France). Lipopolysaccharide (LPS, L2630) was from Sigma-Aldrich (St. Louis, MO). Recombinant mouse CHIL1 (2649-CH-050) was from R&D Systems (Minneapolis, MN).

### Isolation of Mouse Bone Marrow-Derived Macrophages (BMMs)

ICR mice at 4-week-old were sacrificed by cervical dislocation at the time of BMMs harvest. Bone marrow cells were extracted from the tibias and femur by flushing with culture medium using a 25G needle, filter with 70 µm cell strainer (BD Bioscience). Then, the bone marrow cells were harvested by centrifugation at 1200 rpm for 5 min and cultured in RPMI-1640 complete medium containing 10% FBS, 1% penicillin/streptomycin and 10% L929 cell-conditioned medium at 37°C with 5% CO2. Cells were washed with PBS and medium replaced at day 3 and day 5. BMMs were stimulated on day 6 with LPS (10 ng/mL) or alone for 3 hours. Recombinant mouse CHIL1 (2649-CH-050, R&D Systems) stimulated BMMs at 100 μg/mL for 24 hours after LPS.

### Isolation of Mouse Bone Marrow-Derived Neutrophils

Bone marrow-derived neutrophils were obtained from 6-week-old ICR mice as described (23). Bone marrow cells were extracted from the tibias and femur by flushing with PBS using a 25G needle, filter with 70 µm cell strainer. After centrifugation at 1200 rpm for 5 minutes, bone marrow cells suspension was layered on Histopaque 1077 (Sigma-Aldrich) at a ratio of 1:3. After centrifugation at 2000 rpm for 20 minutes (no braking), precipitate was resuspended in PBS. The cell suspension was layered on top of Histopaque 1119 (Sigma-Aldrich) at a ratio of 1:2. After centrifugation at 2000 rpm for 20 minutes (no braking), neutrophils recovered on top of Histopaque 1119. The neutrophils were harvested and washed with PBS, then resuspended in RPMI 1640 medium. Ly6G immunofluorescence staining was used to determine the purity of neutrophils (almost 100% of the cells were positive for Ly6G^+^). Neutrophils were resuspended at a density of 2.5×10^6^ cells/mL and treated with 1 μM S1P for 2 hours (A-Neu) or 0.4% BSA (Vehicle) for 2 hours (N-Neu). The cells were washed with PBS and then resuspended for subsequent animal or cell experiments.

### Animal Models

In this study, 6-week-old male ICR mice were purchased from Charles River in Beijing (Vital River). To induce liver inflammation, mice were fed with control diet or MCDHF diet (A06071309, Research Diet Inc, NJ, USA), an L-amino acid diet containing 45 kcal of fat, 0.1% methionine, and no added choline. After 2 weeks of MCDHF diet, mice were injected once with 5×10^6^ A-Neu or N-Neu, or an equivalent volume of phosphate-buffered saline (PBS) through the tail vein and fed the MCDHF diet continuously. Four days after injection, mice were sacrificed humanely for further analysis. In some cases, neutrophils were obtained from the bone marrow of enhanced green fluorescence protein (EGFP) transgenic mice, 5×10^6^ EGFP^+^ N-Neu, EGFP^+^ A-Neu or PBS were injected into mice fed with MCDHF diet for 2 weeks. The mice were euthanized in 4 hours or 12 hours after cell infusion. Mice were maintained in a specific pathogen free (SPF), temperature-controlled environment with a 12 hours light/dark cycle. The animal studies were performed in compliance with the ethical guidelines for animal studies and approved by the Ethics Committee of Capital Medical University (approval number: AEEI-2017-090).

### Neutrophil/Macrophage Co-Culture

For neutrophil-macrophage co-culture experiments, BMMs were seeded in the lower chamber of the 12-well cell culture plate (Corning, NY, US) at a density of 5×10^5^/well. BMMs were left untreated or converted to pro-inflammatory macrophages by stimulation with LPS (10 ng/mL) for 3 hours, then washed three times with PBS and 1.5 mL RPMI 1640 medium was added. Subsequently, A-Neu or N-Neu were added into the upper chamber of a transwell permeable support with 0.4 μm pore (Corning, NY, US) at a density of 2.5×10^6^/well with 0.5 mL RPMI, to co-culture with pro-inflammatory macrophages. After 6 hours, the macrophages were washed with PBS, and RNA and protein were extracted.

### CHIL1 Immunodepletion

Bone marrow-derived neutrophils were resuspended in the serum-free 1640 medium at a density of 2.5×10^6^ cells/mL, stimulated with 1 μM S1P for 2 hours (A-Neu), then washed with PBS. After further culture in the serum-free 1640 medium for 6 hours at a density of 2.5×10^6^ cells/mL, the cell supernatant was collected (SUP). Protein G-conjugated magnetic beads were incubated with sheep polyclonal anti-CHIL1 antibody (AF2649, R&D Systems). The CHIL1 antibody-conjugated beads were incubated with the cell supernatant obtained from the above on a rotating platform at 4°C overnight, magnetically separated to remove the CHIL1 antibody-conjugated beads-CHIL1 protein complex, and the supernatant (SUP-anti-CHIL1) was collected for subsequent processing of macrophages. BMMs were stimulated with LPS (10 ng/mL) for 3 hours, washed with PBS. And then cultured with 1mL SUP or SUP-anti-CHIL1. After 6 hours, the macrophages were washed with PBS, and RNA and protein were extracted.

### Measurement of Cytokines by Cytometric Bead Array (CBA)

Applied BD CBA Mouse Flex Kit (Catalog No.558266), briefly as follows: the standards (1:2, 1:4, 1:8, 1:16, 1:32, 1:64, 1:128 and 1:256) were prepared by dilution according to the manufacturer’s instructions. liver tissue or cell (60 μg of protein extract) were homogenized to 50 μL. Samples or standards were mixed with TNF (Catalog No. 558299) and CCL4 (MIP-1β, Catalog No. 558343) Capture Beads in 50 μL buffer, and incubated for 1 hour at room temperature. Then 50 μL mixed TNF and CCL4 PE detection reagent was added and incubation was continued for 1 hour at room temperature. After incubation samples were washed with 1 mL of wash buffer, centrifuged at 200×g for 5 min, and then resuspended in 300 μL of wash buffer. The samples were collected on FACS Aria and analyzed by FACP Array V3.

### Flow Cytometry

The mouse liver non-parenchymal cells were isolated as previously described with some modifications (24). Livers were perfused with 20 mL of PBS, minced with scissors, and digested for 30 min with collagenase type IV at 37°C. Digested extracts were filtrated with 70 μm cell strainers to achieve single-cell suspensions. The cell suspension was subjected to density gradient (Histopaque-1077) centrifugation at 2000 rpm for 20 min. After centrifugation, the cells were collected from the interface and temporarily stored at 4°C and labeled as A. The supernatant was discarded below the interface, the precipitated cells were resuspended, and the cell suspension was subjected to density gradient (Histopaque-1119) centrifugation at 2000 rpm for 20 min. After centrifugation, middle layer cells were collected and labeled as B. A and B cells were mixed, washed with PBS twice and resuspended at 1.5×10^6^ cells/100 μL in PBS. Then cells were incubated with the antibody eFlour 450-CD45 (48045182, Thermo Fisher Scientific), APC-CD11b (553312, BD Biosciences), PE-Ly6G (12593182, Thermo Fisher Scientific) and their isotype-matched negative control at 4°C in the dark for 30 min. The cells were washed with PBS and resuspended. Flow cytometry data were acquired by FACS Aria and analyzed by FACS Diva 4.1 (BD, Biosciences).

### Quantitative RT-PCR (RT-qPCR) Analysis

Liver tissue and cells RNA were extracted by Trizol reagent method (Sigma-Aldrich). Total RNA was used for first-strand cDNA synthesis with M-MLV reverse transcriptase (Thermo Fisher Scientific). RT-qPCR was performed utilizing SYBR Green Master Mix (Applied Biosystems) on the ABI 7300 TH Real-Time PCR System (Applied Biosystems). The level of target gene expression was normalized against the GAPDH gene. All primers were synthesized by Biotech (Beijing, China). Primer sequences are listed in **Table 1**.

**Table 1:** Primer sequence.

Mouse	Sequence	
glyceraldehyde-3-phosphate dehydrogenase (*Gapdh*)	Sense	CATGGCCTTCCGTGTTCCTA
	Antisense	GCGGCACGTCAGATCCA
C-C chemokine motif ligand 4 (*Ccl4*)	Sense	CCAGCTCTGTGCAAACCTAACC
	Antisense	GCCACGAGCAAGAGGAGAGA
tumor necrosis factor (*Tnf*)	Sense	GGCAGGTTCTGTCCCTTTCA
	Antisense	CTGTGCTCATGGTGTCTTTTCTG
nitric oxide synthase 2 (*Nos2*)	Sense	TGACGGCAAACATGACTTCAG
	Antisense	GGTGCCATCGGGCATCT
chitinase-like 1 (*Chil1*)	Sense	GTACAAGCTGGTCTGCTACTTC
	Antisense	ATGTGCTAAGCATGTTGTCGC
lymphocyte antigen 6 complex, locus G (*Ly6g*)	Sense	AGAAGCAAAGTCAAGAGCAATCTCT
	Antisense	TGACAGCATTACCAGTGATCTCAGT
adhesion G protein-coupled receptor E1 (*Adgre1*)	Sense	AGCACATCCAGCCAAAGCA
	Antisense	CCATCTCCCATCCTCCACAT
actin alpha 2 (*Acta2*)	Sense	ATGCTCCCAGGGCTGTTTT
	Antisense	TTCCAACCATTACTCCCTGATGT
albumin (*Alb*)	Sense	TGCTTTTTCCAGGGGTGTGTT
	Antisense	TTACTTCCTGCACTAATTTGGCA

### Western Blotting

Proteins were extracted from liver tissues or cells in the presence of protease inhibitors cocktail (Roche Applied Science), resolved by SDS-PAGE and then transferred to PVDF membranes. The membranes were blocked and incubated overnight with primary antibody against NOS2 (1:1000, Cell Signaling Technology) and GAPDH (1:1000, Cell Signaling Technology) at 4°C overnight, then incubated with infrared Dye 800-conjugated secondary antibodies (1:10000, LI-COR Biosciences) for 1 hour at room temperature. Protein level was visualized by the LI-COR Odyssey^®^ Imaging System and assessed by Odyssey^®^ software (LI-COR Biosciences, Lincoln, NE).

### Histology Analysis

Liver tissues were dissected out, further fixed with 4% paraformaldehyde and embedded in paraffin. 5 µm cross sections from fixed liver tissues were stained with hematoxylin-eosin (H&E) for assessment of inflammation as previously described (25). The hepatic inflammatory area was quantified by measuring the area where inflammatory cells were infiltrated using ImageJ 1.4 (NIH). The average of fifteen regions were randomly selected as the inflammatory area in each sample.

### Liver Damage Assessment

Serum alanine aminotransferase (ALT) levels were detected by BS-200 Chemistry Analyzer (Mindray, China) according to the manufacturer’s directions.

### Microarray and Computational Analyses

Neutrophils were treated with or without S1P, and total RNA was collected for further analysis. Converted RNA samples into individual cDNA libraries, and double-stranded cDNA was labeled and hybridized to an Affymetrix Mouse Gene MTA 1.0 array (Affymetrix, Santa Clara, CA, USA). To select the differentially expressed genes, we used threshold values of ≥2-fold change and corrected p value ≤0.05. The function and pathway of differentially expressed genes were explored by GO database. mRNA microarray experiments and technical assistance in bioinformatic analysis were handled by Capitalbio Technology (Beijing, China).

### Statistical Analysis

All analyses were performed with GraphPad Prism 7 (GraphPad, San Diego, CA) and SPSS Statistics 26.0. The results were presented as mean ± SD. Data were determined to be normally distributed in each group using the Shapiro-Wilk test. For statistical significance between every two groups, Student’s t test was applied when data show normal distribution. For multiple group comparison, one-way or two-way ANOVA was used when the data exhibited a normal distribution, while the nonparametric test (Kruskal-Wallis test) was applied for data with a skewed distribution. Correlation coefficients were calculated by Pearson’s test. In all statistical comparisons, a P value of <0.05 was considered as a statistically significant difference. All results were verified in three independent experiments.

## Results

### A-Neu but Not N-Neu Alleviated MCDHF-Induced Liver Injury in Mice

**Figure 1:**
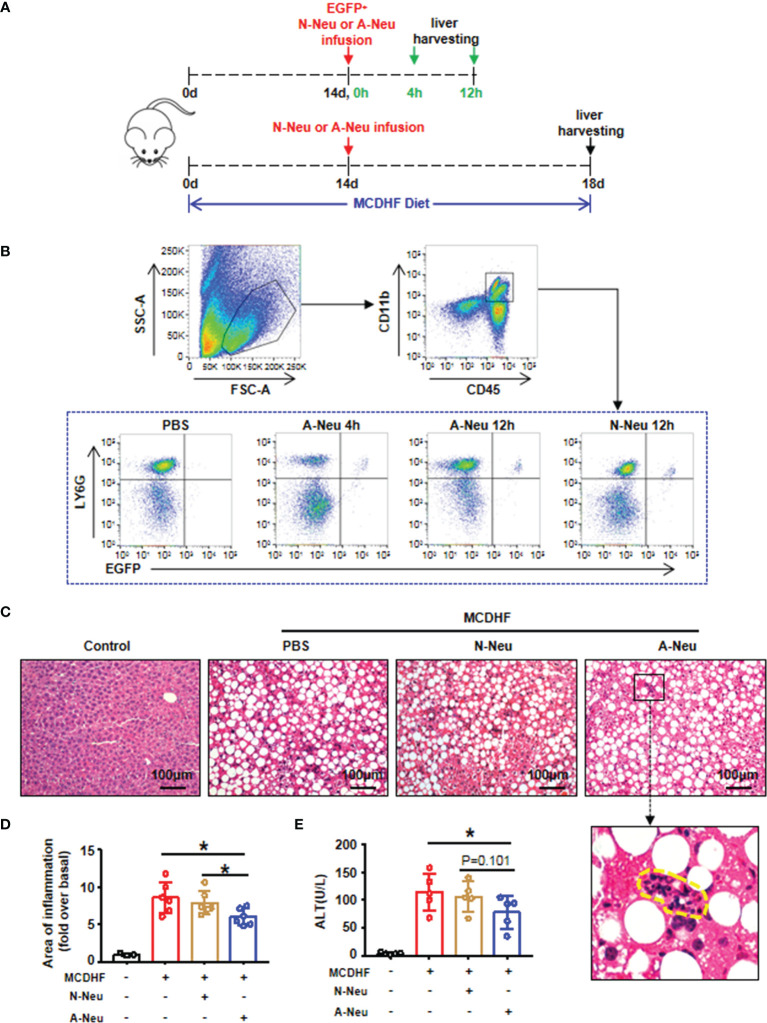
Infusion of A-Neu ameliorated MCDHF-induced liver injury in mice. **(A)** Schematic diagram of experimental design of tail vein injection of neutrophils (5×10^6^ cells/mouse) after 2 weeks of MCDHF diet in mice. **(B)** Representative flow cytometry analysis of EGFP^+^ Ly6G^+^ neutrophils on CD45^+^ CD11b^+^ monocytes in the liver of mice in 4 hours or 12 hours after injection of EGFP^+^ N-Neu or EGFP^+^ A-Neu. **(C)** Infusing N-Neu or A-Neu into mice after 2 weeks of MCDHF diet and harvesting liver in 4 days after injection. The representative H&E staining images of liver sections were performed, inflammatory area was indicated by circle. **(D)** Areas of Inflammation were quantified, (control group, n=3; MCDHF group, n=6). **(E)** Serum ALT levels were measured in indicated groups, n=5 each group. The results were presented as mean ± SD. One-way ANOVA was used. *P < 0.05. Scale bar = 100μm. MCDHF, methionine-choline-deficient and high-fat diet; A-Neu, activated neutrophil; N-Neu, non-activated neutrophil; ALT, alanine aminotransferase; EGFP, enhanced green fluorescence protein.

We analyzed whether activated and non-activated neutrophils affect liver inflammation. As our previous studies have shown that S1P, as a metabolite of cell membrane sphingolipids (26), could change the characteristics of neutrophils, activate neutrophils and prolong their half-life *in vitro* (27). Therefore here we used S1P to activate neutrophils (A-Neu), while neutrophils of bone marrow origin that were not stimulated are called non-activated neutrophils (N-Neu**)**. We first constructed a liver injury model by MCDHF diet for 2 weeks. EGFP^+^ neutrophils were then isolated from the bone marrow of EGFP transgenic mice and stimulated with S1P for 2 hours (A-Neu) or not (N-Neu), and then injected into MCDHF 2-week mice through tail vein. Equal volume of PBS was injected into MCDHF-diet-fed mice as the vehicle group. To observe whether neutrophils injected by tail vein can enter the inflamed liver, the livers were collected in 4 hours or 12 hours after injection, and MCDHF diet was continuous during the period (**Figure 1A**). We isolated hepatic non-parenchymal cells from the mouse livers and detected CD45^+^ CD11b^+^ Ly6G^+^ EGFP^+^ cells by Flow cytometry. At 4 and 12 hours after injection of neutrophils, Ly6G^+^ EGFP^+^ cells (injected neutrophils) could be observed in CD45^+^ CD11b^+^ cells (monocytes), but in the livers of PBS-injected mice, there was no Ly6G^+^ EGFP^+^ cells (**Figure 1B**). These results indicate that neutrophils could be rapidly delivered to the inflamed liver by intravenous injection.

Following the same intravenous regimen described above, mice fed with MCDHF diet for 2 weeks were intravenously infused PBS, N-Neu or A-Neu. Mice were sacrificed in four days after injection, and MCDHF diet was continuous during the period (**Figure 1A**). Liver histology was evaluated by H&E staining (**Figure 1C**) and quantified by digital image analysis (**Figure 1D**). H&E stained sections showed that liver damage was reduced after A-Neu injection in MCDHF mice. Compared with the PBS group and N-Neu group, the inflammatory area of liver decreased significantly after injection of A-Neu (**Figure 1D**). Simultaneously, compared to the PBS group, serum collected from A-Neu group showed significantly decreased levels of ALT (the serum biochemical parameters of liver injury) (**Figure 1E**), indicating that the A-Neu was effective in ameliorating liver injury induced by MCDHF.

### A-Neu Inhibited the Expressions of Pro-Inflammatory Cytokines in the Injured Liver of Mice Treated by MCDHF

**Figure 2:**
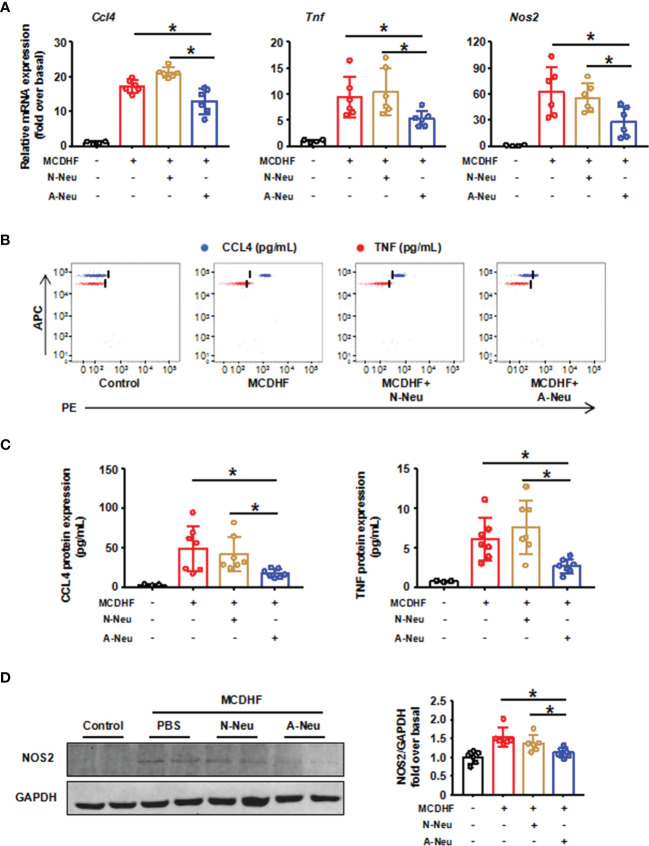
A-Neu inhibited the expression of pro-inflammatory cytokines in the injured liver of mice induced by MCDHF. **(A)** N-Neu or A-Neu were injected into mice 2 weeks after MCDHF diet, and the livers were collected at 4 days after injection. Hepatic expressions of *Ccl4*, *Tnf* and *Nos2* transcripts were measured by RT-qPCR, presented relative to *Gapdh*, (control group, n=4; MCDHF group, n=6). The representative scatter plot of CBA, as shown in **(B)**, and the two bead populations represented CCL4 and TNF respectively. **(C)** Showed the quantification of these protein concentrations (control group, n=3; MCDHF group, n=7). **(D)** NOS2 protein levels in indicated groups were examined by WB (n=6). The results were presented as mean ± SD. One-way ANOVA was used. *P < 0.05. MCDHF, methionine-choline-deficient and high-fat diet; A-Neu, activated neutrophil; N-Neu, non-activated neutrophil; Ccl4, C-C chemokine motif ligand 4; Tnf, tumor necrosis factor; Nos2, nitric oxide synthase 2.

We then asked whether neutrophils could influence the resolution of liver inflammation *in vivo*. **Figure 2A** showed that pro-inflammatory related genes (*Ccl4*, *Tnf* and *Nos2*) mRNA expressions were significantly increased after MCDHF diet compared to the control group. Surprisingly, pro-inflammatory related genes mRNA expressions significantly were reduced in A-Neu group compared with the PBS group and N-Neu group. To further verify this observation, CBA was used to identify CCL4 and TNF protein contents in the liver. And Western Blot (WB) was used to identify NOS2 protein contents. Consistent with the mRNA results, pro-inflammatory cytokines protein expressions also displayed a marked increase in the PBS group compared with the control group. Moreover, the content of these proteins in A-Neu group were decreased significantly, compared with the PBS group and N-Neu group (**Figures 2B**–**D**). These results suggest that A-Neu can inhibit the expressions of pro-inflammatory markers in the injured liver of mouse induced by MCDHF, whereas N-Neu do not have this effect.

### A-Neu Inhibited the Pro-Inflammatory Phenotype of Macrophages *In Vitro*

**Figure 3:**
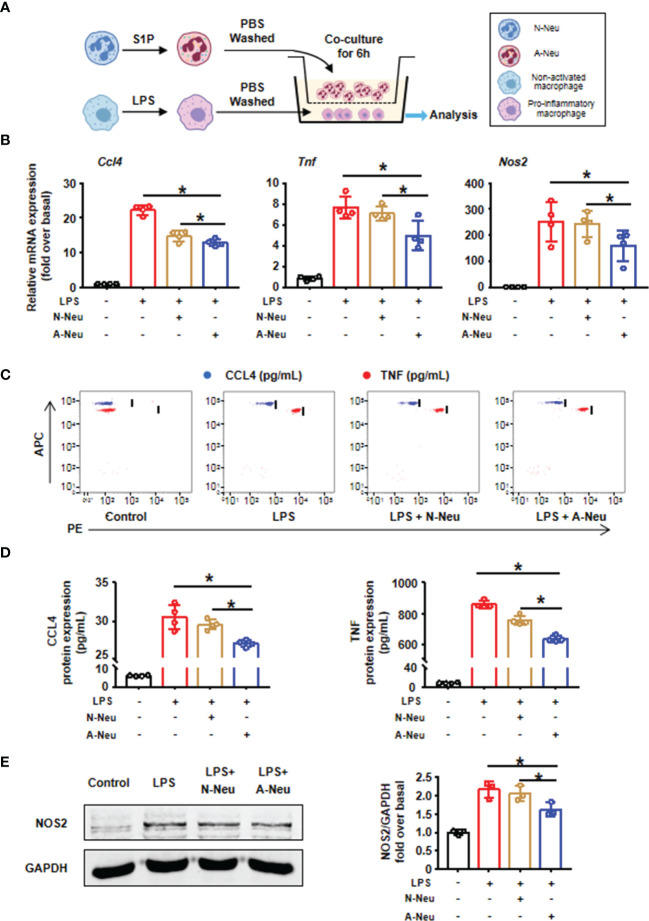
A-Neu inhibited the pro-inflammatory phenotype of macrophages *in vitro*. **(A)** Schematic representation of the experimental design: bone marrow-derived macrophages were polarized into pro-inflammatory phenotype with LPS 10 ng/mL for 3 hours, and then PBS washed the cells, at 6 hours after the co-culture with N-Neu or A-Neu, macrophages were harvested. **(B)** The mRNA expression levels of genes *Ccl4*, *Tnf* and *Nos2* were evaluated using RT-qPCR, presented relative to *Gapdh*, (n=4). The protein expressions of CCL4, TNF and NOS2 in liver were detected by CBA **(C, D)**, n=4, and WB **(E)**, n=3. The results were presented as mean ± SD. One-way ANOVA was used. *P < 0.05. A-Neu, activated neutrophil; N-Neu, non-activated neutrophil; Ccl4, C-C chemokine motif ligand 4; Tnf, tumor necrosis factor; Nos2, nitric oxide synthase 2; LPS, lipopolysaccharide.

To explore whether the inhibitory effect of A-Neu on hepatic inflammation was mediated through communication with macrophages, BMMs were polarized into pro-inflammatory phenotype with LPS, the expressions of pro-inflammatory related genes (*Ccl4*, *Tnf* and *Nos2*) increased significantly at the mRNA level (**Figures 3A, B**). Subsequently, to investigate the role of neutrophils in macrophage inflammation, we prepared a neutrophil and macrophage co-culture system. After pro-inflammatory polarization with LPS, BMMs were co-cultured with bone marrow-derived N-Neu or A-Neu, and then the expressions of macrophage pro-inflammatory markers were examined (**Figure 3A**). Excitingly, when pro-inflammatory macrophages were co-cultured with A-Neu, the expressions of pro-inflammatory related genes (*Ccl4*, *Tnf* and *Nos2*) at the mRNA level were significantly reduced, and compared with the N-Neu group, the expressions of pro-inflammatory related genes were also obvious reduced (**Figure 3B**). Similar results were observed in pro-inflammatory cytokine protein levels of LPS treated macrophages examined by CBA and WB (**Figures 3C**–**E**). Collectively, these results demonstrate that A-Neu significantly suppress pro-inflammatory macrophage responses *in vitro*.

### A-Neu Secreted CHIL1, Which Was Positively Correlated With Neutrophil Marker Ly6G in the Injured Liver of Mice

**Figure 4:**
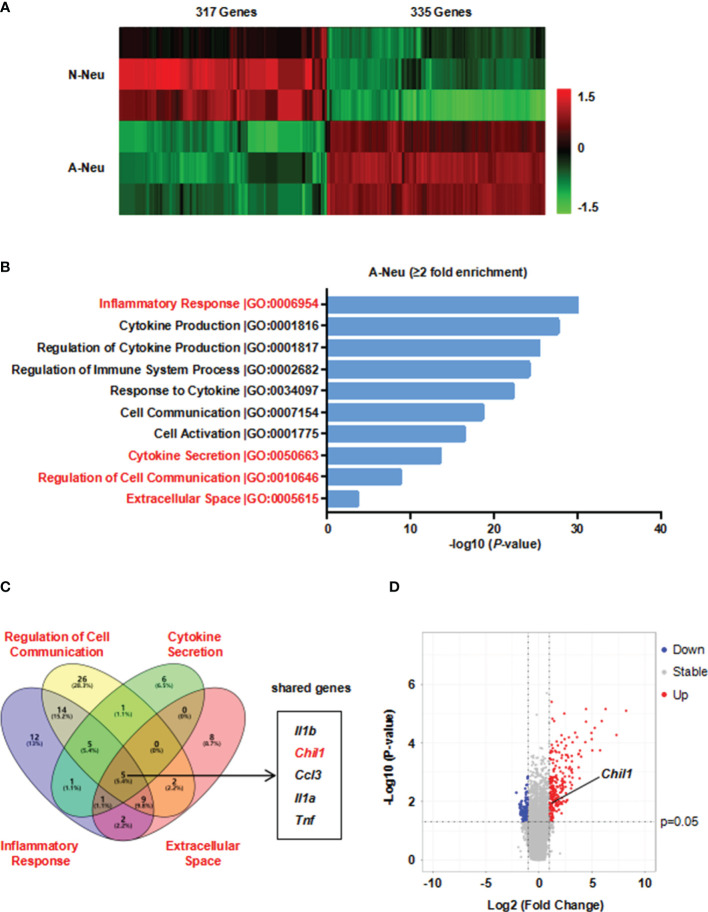
Microarray analysis was performed in N-Neu and A-Neu. **(A)** Microarray heatmap data showing the expression level of transcript between N-Neu and A-Neu. The mRNA expressions were expressed as follows: Bright green, under expression; black, no change; bright red, overexpression. n=3. Row-based Z-score normalized. **(B)** GO enrichment analysis of differentially expressed genes in S1P activated neutrophils. **(C)** Venn diagram of gene expression overlap between GO terms including Regulation of Cell Communication, Cytokine Secretion, Inflammatory Response and Extracellular Space clusters. **(D)** Volcano plot of A-Neu gene expression compared with N-Neu. The threshold values were fold change≥|2| and P value ≤ 0.05. Red dots and blue dots represent up-regulated and down-regulated genes, respectively. Gray dots indicate genes with no statistically significant differences. The arrow indicated the location of Chil1. A-Neu, activated neutrophil; N-Neu, non-activated neutrophil; Il1b, Interleukin 1 beta; Chil1, Chitinase-like 1; Ccl3, Chemokine (C-C motif) ligand 3; Il1a, Interleukin 1 alpha; Tnf, tumor necrosis factor.

To investigate the mechanism by which A-Neu inhibiting pro-inflammatory macrophage responses, we performed a microarray experiment using the RNA of N-Neu and A-Neu, and we explored mRNA expression profiles (**Supplementary Table 1**). Hierarchical clustering showed systematic variations in transcript expression levels between N-Neu and A-Neu, with 335 mRNAs upregulated and 317 mRNAs downregulated after S1P activation (**Figure 4A**). Gene Ontology (GO) enrichment analysis revealed that S1P activation affected a list of genes associated with Inflammatory Response, Cytokine Production, Regulation of Cytokine Production, Regulation of Immune System Process, Response to Cytokine, Cell Communication, Cell Activation, Cytokine Secretion, Regulation of Cell Communication, Extracellular Space, etc (**Supplementary Table 2** and **Figure 4B**).

We hypothesized that the factors released by A-Neu may communicate cellularly with macrophages, thereby suppressing pro-inflammatory macrophage responses. To verify this possibility, we used Gene Venn software to show the gene expression overlap between GO terms including Regulation of Cell Communication, Cytokine Secretion, Inflammatory Response and Extracellular Space clusters, and obtained 5 shared genes, including *Il1b*, *Chil1*, *Ccl3*, *Il1a* and *Tnf* (**Figure 4C**). Among them, Chil1 (Chitinase-like 1), also named Chi3l1, a highly evolutionarily conserved secreted protein. CHIL1 is synthesized and secreted by a multitude of cells including macrophages, neutrophils and other cells (28). Numerous studies have demonstrated that CHIL1 can inhibit the inflammatory phenotype of macrophages (29–31). Taken together, CHIL1 secreted by neutrophils after S1P activation may be a key effector in inhibiting macrophage inflammatory response. Volcano plot represented the distribution of the fold changes and P-values of A-Neu compared with N-Neu, as shown in **Figure 4D**.

**Figure 5:**
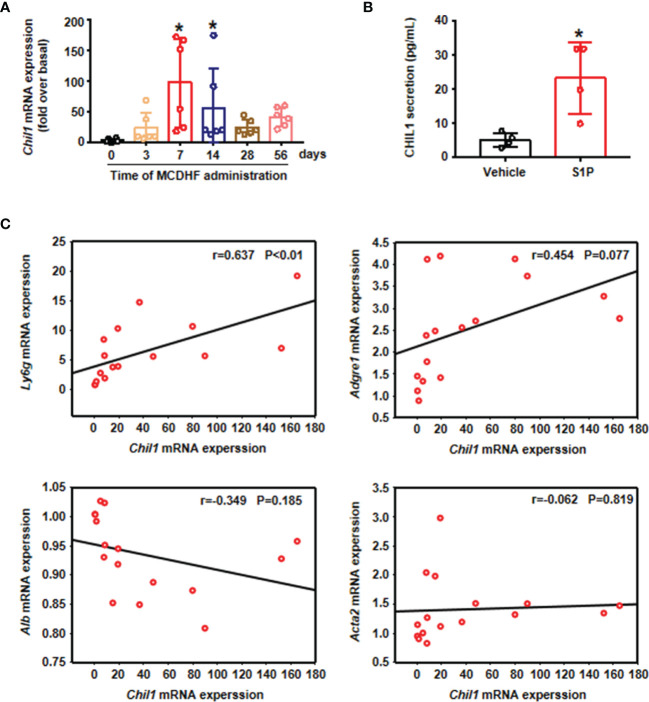
*Chil1* was positively correlated with *Ly6g* in the injured liver of mice and could be secreted by A-Neu. **(A)** The mRNA expression of Chil1 was examined by RT-qPCR in the injured liver of MCDHF-treated mice in indicated times (n=6). **(B)** S1P stimulated neutrophils for 2 hours, then PBS washed the cells, and continued to culture for 6 hours, the secretion of CHIL1 in cell supernatant was detected by ELISA, n=4. **(C)** Correlation analysis between *Chil1* mRNA expression with neutrophil marker *Ly6g*, macrophage marker *Adgre1*, hepatocyte marker *Alb* and myofibroblast marker *Acta2* in the injured liver of MCDHF-treated mice. Nonparametric test (Kruskal-Wallis test) was used in **(A)** Student’s t test was used in **(B)** Pearson’s test was used in **(C)** *P < 0.05 vs. MCDHF treated group for 0 days or control. MCDHF, methionine-choline-deficient and high-fat diet; S1P, Sphingosine 1-phosphate; Chil1, Chitinase-like 1; Ly6G, lymphocyte antigen 6 complex, locus G; Adgre1, adhesion G protein-coupled receptor E1; Acta2, actin alpha 2; Alb, albumin.

To assess the functional role of CHIL1, we investigated the dynamic changes of *Chil1* mRNA expression in the liver of mice treated with MCDHF diet at different time points. The results showed that *Chil1* mRNA expressions were upregulated from 7 days after treated with MCDHF diet, and then dramatically decreased after 14 days (**Figure 5A**). Then we undertook correlation analysis between *Chil1* mRNA expression and neutrophil marker *Ly6g* mRNA expression in MCDHF-fed mice. We discovered that hepatic *Chil1* expression positively correlated with *Ly6g* mRNA expression. However, there was no significant correlation with macrophage marker *Adgre1*, hepatocyte marker *Alb*, and myofibroblast marker *Acta2* (**Figure 5C**). Simultaneously, after S1P activation of neutrophils, the secretion of CHIL1 in the cell supernatant was significantly increased (**Figure 5B**). These results indicate that Chil1 is strongly associated with neutrophils, and activation of neutrophils by S1P can induce the secretion of CHIL1.

### Neutrophil-Derived CHIL1 Inhibited Pro-Inflammatory Macrophage Responses

**Figure 6:**
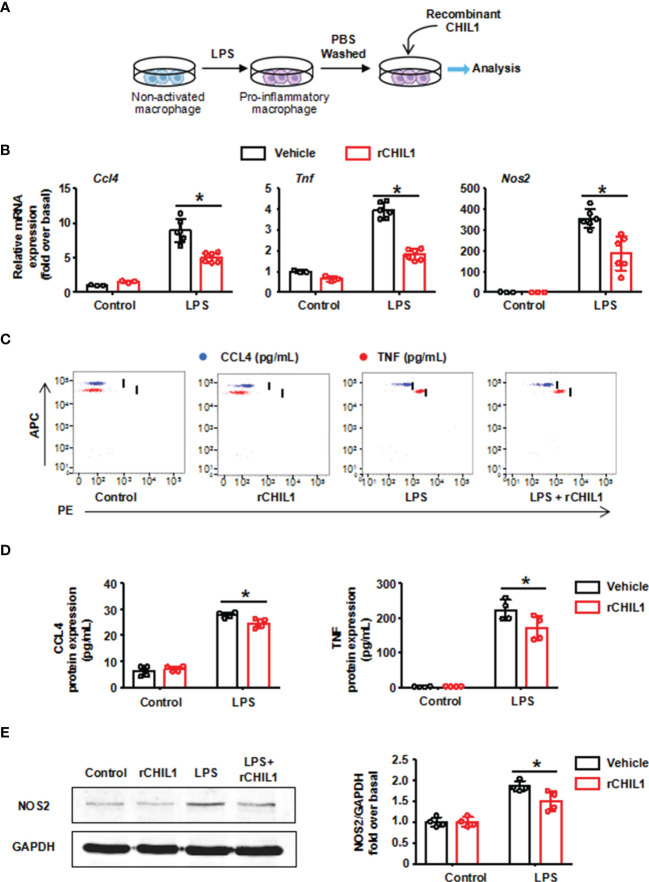
Recombinant CHIL1 inhibited the pro-inflammatory phenotype of macrophages. **(A)** Schematic representation of the experimental design: bone marrow-derived macrophages were stimulated with LPS 10 ng/mL for 3 hours, washed with PBS, and then treated with Vehicle (PBS) or rCHIL1 (100 ng/mL) for 24 hours respectively. **(B)** Macrophages were harvested and the mRNA expression levels of genes *Ccl4*, *Tnf* and *Nos2* were evaluated using RT-qPCR, presented relative to *Gapdh*, (control group, n=3; LPS group, n=6). The protein expressions of CCL4, TNF and NOS2 in liver were detected by CBA **(C, D)** and WB **(E)** n=4. The results were presented as mean ± SD. Two-way ANOVA was used. *P < 0.05. rCHIL1, Recombinant CHIL1; Ccl4, C-C chemokine motif ligand 4; Tnf, tumor necrosis factor; Nos2, nitric oxide synthase 2; LPS, lipopolysaccharide.

In order to further verify whether CHIL1 secreted by A-Neu is a key effector that affects the phenotype of macrophages, we used recombinant CHIL1 (rCHIL1) to culture LPS-induced pro-inflammatory macrophages (**Figure 6A**). Strikingly, the effect of A-Neu on macrophage phenotype was reproduced by incubating *in vitro* pro-inflammatory macrophages with rCHIL1. The mRNA expressions of pro-inflammatory related genes (*Ccl4*, *Tnf* and *Nos2*) in macrophages were significantly decreased after treatment with rCHIL1 (**Figure 6B**). **Figures 6C**–**E** showed that the expressions of these pro-inflammatory cytokines were also decreased at the protein level.

**Figure 7:**
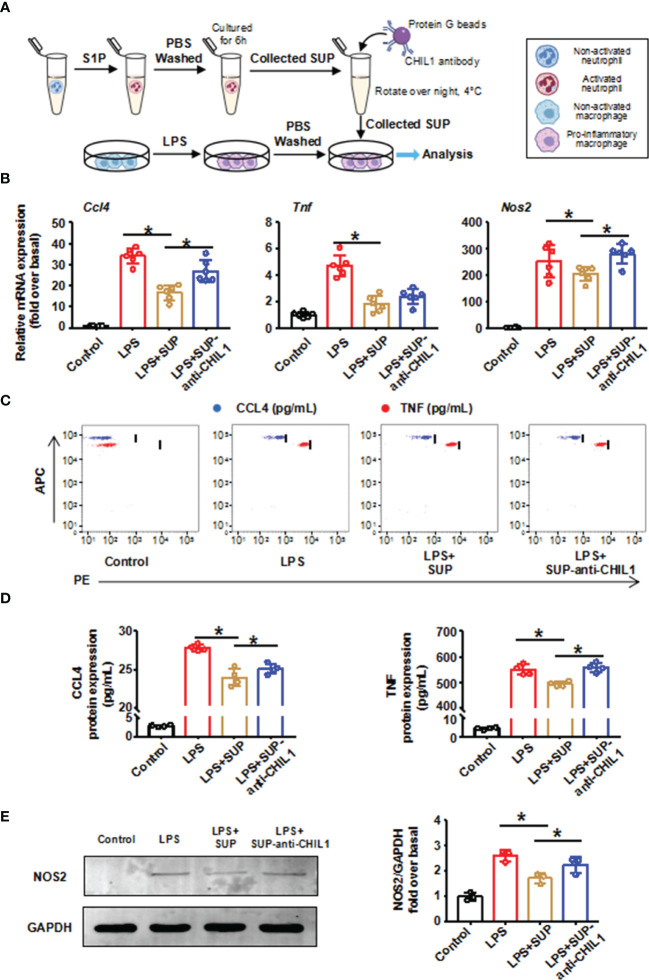
Immunodepletion of CHIL1 in A-Neu supernatant attenuated the inhibitory effect on pro-inflammatory macrophages. **(A)** Schematic representation of the experimental design: bone marrow-derived macrophages were stimulated with LPS 10 ng/mL for 3 hours, washed with PBS, and then cultured in the presence of A-Neu supernatant (SUP) or A-Neu supernatant immunodepleted with anti-CHIL1 (SUP-anti-CHIL1). Expressions of CCL4, TNF and NOS2 in indicated groups were detected by RT-qPCR (**B**, n=6), CBA (**C, D**, n=4) and WB (**E**, n=3). The results were presented as mean ± SD. One-way ANOVA was used. *P < 0.05. A-Neu, activated neutrophil; N-Neu, non-activated neutrophil; SUP, activated neutrophil supernatant; SUP-anti-CHIL1, activated neutrophil supernatant immunodepletion CHIL1; Ccl4, C-C chemokine motif ligand 4; Tnf, tumor necrosis factor; Nos2, nitric oxide synthase 2; LPS, lipopolysaccharide.

To validate this finding, we performed immunodepletion of CHIL1 in A-Neu supernatant. First, BMMs were induced to a pro-inflammatory phenotype with LPS, and then cultured in the presence of A-Neu supernatant (SUP) or A-Neu supernatant immunodepletion CHIL1 with protein G-conjugated magnetic beads-CHIL1 antibody (SUP-anti-CHIL1). Consistent with **Figure 2** results, the expressions of pro-inflammatory related genes (CCL4, TNF and NOS2) at the mRNA and protein levels were significantly decreased when pro-inflammatory macrophages were cultured with SUP. However, when pro-inflammatory macrophages were cultured with SUP-anti-CHIL1, the inhibitory effects on pro-inflammatory macrophage were partially blunted (**Figures 7A**–**E**). Altogether, these data suggest that CHIL1 secreted by A-Neu are important regulators in inhibiting the response of pro-inflammatory macrophages.

## Discussion

**Figure 8:**
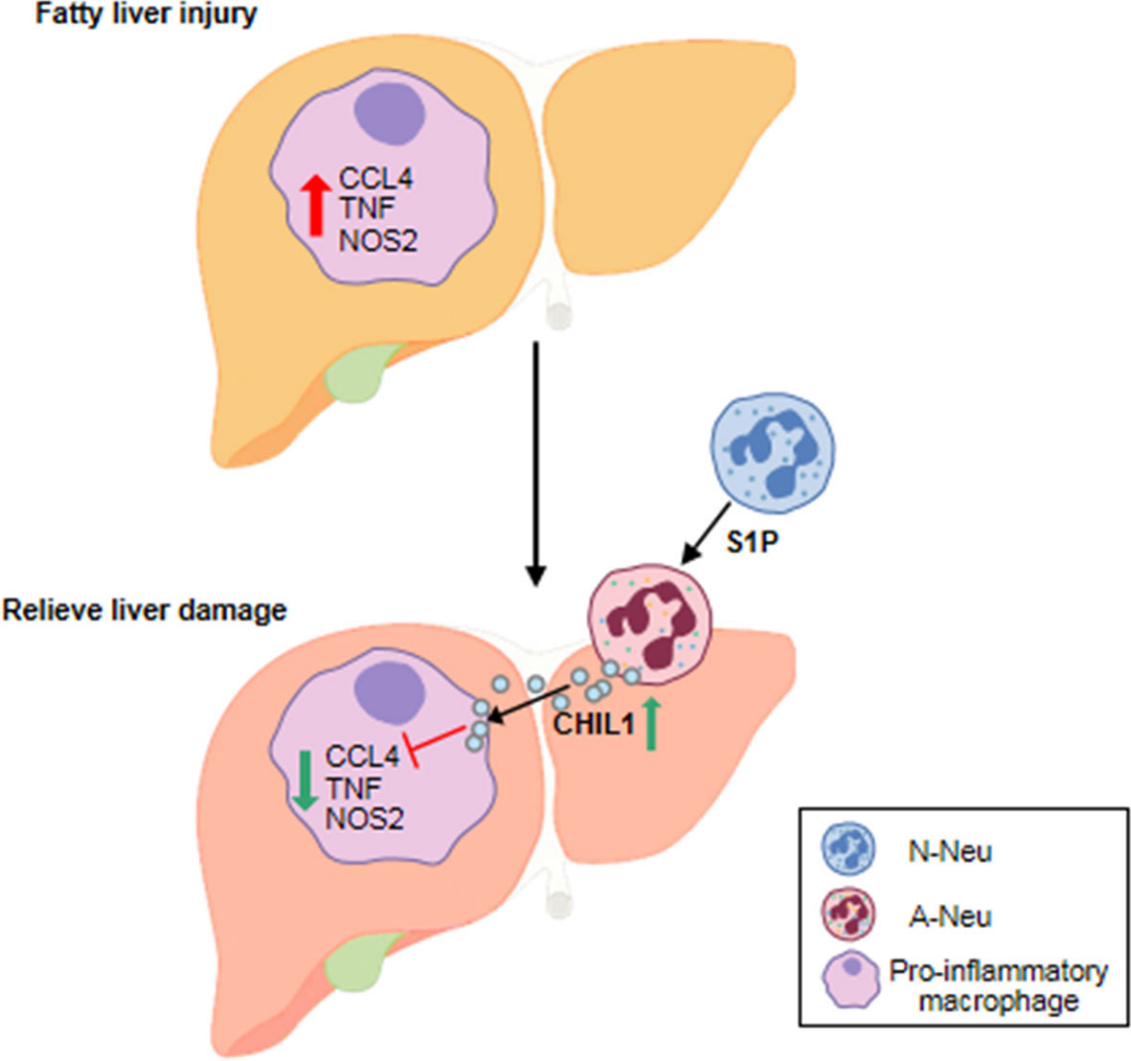
The schematic diagram of the main content of this study. A-Neu secrete CHIL1 and reduce liver inflammation by inhibiting the pro-inflammatory macrophage response. A-Neu, activated neutrophil; N-Neu, non-activated neutrophil; Ccl4, C-C chemokine motif ligand 4; Tnf, tumor necrosis factor; Nos2, nitric oxide synthase 2; S1P, Sphingosine 1-phosphate; Chil1, Chitinase-like 1.

In liver injury, the classical idea is that neutrophil recruitment and activation are detrimental and will further exacerbate tissue damage (32–34). In this study, our investigations revealed a previously overlooked beneficial role of A-Neu in liver inflammation. A concept diagram was drawn to summarize our main findings (**Figure 8**). In MCDHF-induced fatty liver injury, the number of pro-inflammatory macrophages increases, exacerbating liver injury and fibrosis (35). By infusing A-Neu, the expressions of inflammatory cytokines in liver were effectively suppressed and liver injury was attenuated. A-Neu significantly suppressed the pro-inflammatory phenotype of macrophages, which was mediated by the secreted factor CHIL1. A-Neu played an important role in the resolution of inflammation after tissue injury through crosstalk with macrophages.

Outdated orthodoxy has been challenged by new evidence that neutrophils are a homogeneous cell population. More and more evidence show that different subpopulations of neutrophils have different immunomodulatory effects under homeostasis and disease conditions (36). In this study, we noted that there was functional heterogeneity between A-Neu and N-Neu. A-Neu affected a series of genes related to Inflammatory Response, Cytokine Production and Cell Communication, etc. Moreover, A-Neu significantly suppressed the pro-inflammatory response of macrophages compared to N-Neu. A recent study showed that compared with non-activated neutrophils from bone marrow, activated neutrophils from liver of mouse treated with APAP for 24-hour resulted in reduced expression of pro-inflammatory markers and increased expression of pro-resolving markers in macrophages (20). Wound neutrophils isolated from the s.c. polyvinyl alcohol sponge wound model also played a similar role in regulating the phenotype of macrophages (37). These evidences support our findings to some extent. Compared with the previous study, we injected A-Neu or N-Neu into liver injury mice induced by MCDHF diet for 2 weeks through tail vein, which further proved that A-Neu provide the possibility to limit the progress of inflammation in inflammatory disease states, especially in the case of neutropenia. After feeding MCDHF diet for 2 weeks, the liver inflammation of mice continued to aggravate (35), but the endogenous neutrophils began to decrease (27). Supplementation of A-Neu at this time point could inhibit the progress of inflammation.

S1P has been shown to be involved in a variety of important biological processes, including cell activation, proliferation, apoptosis, chemotaxis, differentiation and regulation of immune function (38–40). Our previous results have documented that S1P significantly activated neutrophils *in vitro*, prolonging their half-life and delaying apoptosis (27). In addition, endogenous S1P levels were significantly elevated in human injured livers caused by multiple etiologies (41), as well as in mouse models including MCDHF diet (35), bile duct ligation and carbon tetrachloride (42). In the absence of intervention, neutrophils recruited to the damaged liver were in the microenvironment of S1P high concentration, allowing neutrophils to be activated, which may limit macrophage inflammation to a certain extent. However, unlike neutrophils activated by S1P, N-Neu injected into the damaged liver did not exert anti-inflammatory effects. This may be due to the fact that neutrophils activated by S1P *in vitro* have a stronger biological effect and do not require additional time for activation after being injected into mice to exert a direct inhibitory effect on inflammation, suggesting that A-Neu may be applied as a cytotherapy for inflammatory diseases.

CHIL1 is commonly elevated in the serum and tissues of patients with a variety of chronic inflammatory diseases, including rheumatoid arthritis, systemic lupus erythematosus (43), and inflammatory bowel disease (44). Elevated levels of circulating CHIL1 were also shown in liver disease characterized by liver inflammation and fibrosis (45). Similarly, we also demonstrated that the expression of CHIL1 was increased in MCDHF-induced fatty liver injury. In our study, liver inflammation was significantly suppressed and liver injury was ameliorated by injection of CHIL1 highly secreted neutrophils. A recent evidence suggested that rCHIL1 administration inhibited the proliferation and function of activated T cells and attenuated liver injury in a Concanavalin A-induced mouse model of liver injury (46). However, it has also been shown that CHIL1 deficiency could protect against ethanol-induced liver injury (47). This may be due to the different roles of CHIL1 in liver injury caused by different etiologies and pathogenesis. Therefore, the role of CHIL1 in liver diseases with different causes and mechanisms needs to be further studied. In our study, the expression of CHIL1 in mouse liver increased from 7 days to 14 days after MCDHF feeding, and decreased significantly after 14 days, but the liver injury continued to worsen. Supplementing CHIL1-high-expressing neutrophils at the time point of MCDHF-14 days significantly improved the progress of inflammation.

Neutrophils are the first responders after tissue injury and one of the first leukocytes to reach the inflammatory site (48). Taking advantages of these characteristics of neutrophils, many studies have tried to use neutrophils as a delivery system for drugs or loaded nanocarriers (49–51). Intravenous injection of drug loaded neutrophils could effectively reduce the level of inflammatory cytokines in local tissues of inflammatory skeletal muscle or myocardial ischemia-reperfusion injury mouse models (49). Here, we also exploited the ability of neutrophils to rapidly reach the site of inflammation, and the recruitment of injected neutrophils into the damaged liver within 4 hours could be observed by tail vein injection of EGFP^+^ neutrophils. Because excessive neutrophils may lead to aggravated inflammation, studies have shown that in a mouse model of LPS-induced skeletal muscle damage, the administration of 10^7^ neutrophils per mouse by intravenous injection did not cause additional inflammation (49). In our study, the injection of 5×10^6^ neutrophils per mouse through the tail vein also did not cause an exacerbated inflammatory response. But if more neutrophils need to be injected, the pro-inflammatory effect of neutrophils needs to be assessed.

In conclusion, our results demonstrated that A-Neu inhibited the inflammatory response of macrophages by releasing CHIL1, thereby ameliorating liver inflammation, which provides a new idea for the treatment of inflammatory liver diseases.

## Data Availability Statement

The original contributions presented in the study are included in the article/**Supplementary Material**. Further inquiries can be directed to the corresponding author.

## Ethics Statement

The animal study was reviewed and approved by Ethics Committee of Capital Medical University.

## Author Contributions

LL conceived and designed the study. YL designed the research studies, conducted experiments, acquired data and analyzed data. YL and LL drafted the manuscript. NC, XZ, RX, and JL conducted experiments and acquired data. LY provided reagents. All authors contributed to the article and approved the submitted version.

## Funding

This work was supported by grants from the National Natural and Science Foundation of China (81970532, 81670550).

## Conflict of Interest

The authors declare that the research was conducted in the absence of any commercial or financial relationships that could be construed as a potential conflict of interest.

## Publisher’s Note

All claims expressed in this article are solely those of the authors and do not necessarily represent those of their affiliated organizations, or those of the publisher, the editors and the reviewers. Any product that may be evaluated in this article, or claim that may be made by its manufacturer, is not guaranteed or endorsed by the publisher.

## Abbreviations

MCDHF diet, methionine-choline-deficient and high-fat diet; BMM, bone marrow-derived macrophage; S1P, Sphingosine 1-phosphate; LPS, lipopolysaccharide; A-Neu, activated neutrophils; N-Neu, non-activated neutrophils; Chil1, Chitinase-like 1; rCHIL1, Recombinant CHIL1; APAP, Acetaminophen; Ccl4, C-C chemokine motif ligand 4; Tnf, tumor necrosis factor; Nos2, nitric oxide synthase 2; Ly6G, lymphocyte antigen 6 complex, locus G; Adgre1, adhesion G protein-coupled receptor E1; Acta2, actin alpha 2; Alb, albumin; ALT, alanine aminotransferase; EGFP, enhanced green fluorescence protein.
