# Supplementary material for: Activated Neutrophils Secrete Chitinase-Like 1 and Attenuate Liver Inflammation by Inhibiting Pro-Inflammatory Macrophage Responses
Source: Front Immunol. 2022 Apr 21;13:824385. doi: 10.3389/fimmu.2022.824385 (PMC9069964; doi:10.3389/fimmu.2022.824385)
Supplement: Supplementary file 1 [file Table_1.pdf]

Supplementary table 1. The preliminary probe signal values of genes by microarray analysis in S1P-activated neutrophils

Probe Set ID	Gene Symbol	Ctrl-1	Ctrl-2	Ctrl-3	S1P-1	S1P-2	S1P-3
TC1400001182.mm.1	Irg1	190.7079	129.0048	97.60024	39741.94	40196.03	39589.81
TC1700001954.mm.1	Tnf	632.3313	558.3027	252.5018	86614.04	108582.5	93517.77
TC1100003401.mm.1	Ccl3	548.5966	520.3871	379.9724	32635.19	42019.58	42179.24
TC1000001813.mm.1	Tnfaip3	130.0477	98.24356	78.12527	5188.812	7006.096	6094.539
TC0200004602.mm.1	Il1b	10591.95	7253.229	3769.495	369676.3	375266.3	374836.9
TC1100002771.mm.1		210.5384	169.9541	93.15764	5844.868	8854.71	6713.257
TC0500000850.mm.1	Cxcl2	91.8009	96.80417	73.99174	3522.553	5922.353	3667.116
TC1200001768.mm.1	Nfkbia	1361.03	925.4655	602.8004	22477.68	30353.92	29873.61
TC0900000184.mm.1	Icam1	82.69215	63.28156	69.92896	1714.033	3310.906	2173.497
TC0600003084.mm.1	Clec4e	881.0541	835.1638	516.9919	21096.95	26938.76	26233.54
TC0900003125.mm.1	Ccrl2	61.86127	54.51287	49.50132	1087.412	1595.197	1190.358
TC0200004599.mm.1	Il1a	71.67001	64.32384	76.62049	1274.808	1711.961	1574.348
TC1600001742.mm.1	Nfkbiz	230.3256	179.4194	130.9661	2947.921	4128.577	3729.958
TC1100002565.mm.1	Mir146	41.28609	24.78803	28.03035	299.4544	502.6511	449.2242
TC0900001723.mm.1		347.8457	208.9478	99.73283	2671.417	3523.599	2988.224
TC1900000497.mm.1	Fas	160.3381	125.2989	89.03091	1374.425	1895.475	1782.143
TC1500002316.mm.1	Gpr84	331.5361	246.8134	94.26417	3367.378	3719.599	3452.078
TC1800001197.mm.1	Cd14	199.3823	226.1321	129.5241	2091.281	3335.082	2780.511
TC0400003957.mm.1	Tnfrsf1b	978.4283	658.038	420.8027	6477.111	9003.65	8497.114
TC0200003209.mm.1	Slc2a6	358.4275	304.1403	194.8302	2930.788	3957.712	3906.063
TC1100002770.mm.1	Tnip1	124.2612	104.0398	87.52241	1059.826	1506.748	1257.47
TC1800000929.mm.1	Map3k8	131.5458	137.0998	116.5822	1095.57	1614.225	1434.367
TC0500003243.mm.1	Hcar2	265.7469	314.0498	223.9223	1831.104	2352.615	2468.307
TC0400003522.mm.1	Zc3h12a	331.6922	255.2405	134.7688	1715.221	2604.907	2226.037
TC1000003019.mm.1	Irak3	614.2698	453.2624	285.3336	2828.935	3791.945	3932.073
TC1900000441.mm.1	Cd274	42.79673	58.38885	43.97498	300.2034	532.8218	365.2859
TC1100000726.mm.1	Nlrp3	1095.982	671.5148	379.3487	4619.506	6983.125	5777.395
TC0100003053.mm.1		156.9052	283.5503	108.835	964.3411	1414.682	1441.412
TC0900001142.mm.1	Bcl2a1a	62.81137	94.06996	108.9008	663.2054	870.4907	801.2977
TC1000000877.mm.1	Gadd45b	208.501	213.4389	102.4381	1542.699	2201.842	1769.438
TC0300001937.mm.1	Slc7a11	294.8919	242.9046	143.9909	1478.497	2108.823	1941.965
TC1700000505.mm.1	Pim1	823.4893	547.5839	265.2836	3487.775	5195.082	4146.985
TC0900002295.mm.1		94.05954	103.9501	77.21951	475.4907	1043.739	794.0506
TC0800002634.mm.1	N4bp1	1358.049	1218.35	469.5312	9160.339	10028.36	10067.75
TC1700001034.mm.1	Adgre1	370.3468	302.9709	186.3347	1925.284	2503.102	2212.393
TC0200003478.mm.1		836.7305	566.7356	224.7148	3193.141	6274.77	3829.013
TC0400001510.mm.1	Rnf19b	526.6814	369.9413	240.8241	2194.115	3212.09	2597.306
TC1700001522.mm.1	Fpr1	10054.96	8925.618	4978.562	56520.39	76713.86	69071.91
TC1700000864.mm.1	Nfkbie	62.56538	59.76917	50.13425	373.9277	517.4559	420.0859
TC0300001938.mm.1		86.4726	59.50437	59.76344	343.2779	519.8652	398.3039
TC1200001237.mm.1	Tnfaip2	476.5934	369.1103	293.7949	2088.7	2748.791	2559.621
TC0600003309.mm.1		62.30183	67.48013	49.48324	536.4564	342.3809	409.8202
TC0200003475.mm.1		100.025	174.9863	61.31372	494.355	849.6312	673.4214
TC0100001338.mm.1	Ptgs2	51.69496	60.62399	43.71818	309.14	476.5483	338.2133
TC0100003051.mm.1	Mapkapk2	2871.39	1833.585	743.4995	9412.282	13059.06	10695.34
TC1900000498.mm.1	FAS-AS1	111.8128	118.1195	150.8135	633.9761	865.3381	747.418
TC0200003793.mm.1	Gm24996	807.7198	609.6852	251.3661	2248.484	3771	3601.625
TC0600001397.mm.1	Clec4a1	86.66849	90.12676	77.04094	504.8889	675.7384	457.7784
TC1900000689.mm.1	Nfkb2	99.75893	89.04787	73.80743	376.0764	581.2142	522.9306
TC0900001722.mm.1	Birc3	306.6462	286.8002	190.4789	1590.066	1886.555	1746.068
TC0400001661.mm.1	Ifnlr1	442.8629	257.9238	122.0961	1162.067	1822.229	1425.166
TC1000000778.mm.1	Icosl	316.6166	299.7289	169.489	1627.354	1957.724	1816.56
TC0600002142.mm.1	Clec5a	1895.31	1627.874	576.0593	8678.649	10866.96	10269.97
TC0200003791.mm.1	Nfe2l2	1076.674	654.5228	438.8993	3244.662	3913.232	3796.543
TC1500001659.mm.1		84.42769	65.27496	48.63869	299.4562	483.4104	348.7639
TC1700000673.mm.1	Ltb	702.1581	676.775	322.9718	3070.417	4716.608	3826.361
TC0200003477.mm.1		250.3825	202.9	127.3214	838.0872	1307.188	1092.578
TC0900002197.mm.1	Il10ra	154.4016	151.9284	96.42997	647.6636	1210.189	810.9694
TC1500000038.mm.1		300.6133	310.5905	143.5254	1602.421	1711.358	1677.977
TC1000002325.mm.1		185.328	232.1	112.4286	774.0766	958.306	1240.075
TC1500002318.mm.1	Itga5	122.0324	113.2993	111.0335	496.6429	744.5259	617.8359
TC0200004094.mm.1		568.713	461.9537	204.2752	2135.698	3129.237	2578.253
TC0500002756.mm.1	Cxcl10	31.41128	29.42952	34.10206	133.6808	167.8539	177.5122
TC1700000148.mm.1	Sod2	171.6242	147.3259	157.2868	788.3696	828.5811	903.4277

TC0200004092.mm.1		365.5852	218.8723	115.8959	1050.882	1805.308	1155.285
TC0200004091.mm.1		1031.641	898.0493	325.3182	3660.509	4946.221	5016.907
TC0900003331.mm.1	Bcl2a1b; Bcl2a1a	129.4502	306.8853	140.1266	791.488	573.2108	690.2417
TC0300002995.mm.1		363.877	273.7849	148.8323	1120.369	1282.327	1484.394
TC0400001511.mm.1		112.8764	105.6449	81.54586	534.7269	570.8636	450.9894
TC0200003476.mm.1		507.5217	592.498	195.0078	1886.859	4604.86	2546.04
TC0600001404.mm.1	Clec4d	7213.008	5642.33	3331.785	25330.81	26524.57	27183.6
TC0200000376.mm.1	Il1f9	74178.47	53284.47	31599.35	210888.5	260658.3	248938.9
TC0200000382.mm.1	Il1rn	1331.16	1168.196	758.3685	4403.244	5750.803	5534.977
TC1100002664.mm.1	Sqstm1	2431.203	1955.439	1057	8100.2	10912.37	8958.223
TC0200000834.mm.1		315.0878	177.3633	163.1141	737.8079	878.8193	826.4575
TC1500001067.mm.1	Nr4a1	188.8434	154.5284	101.1754	501.9418	727.9897	692.6518
TC1100000639.mm.1	Gm12230	51.33668	49.87906	57.66872	173.5937	342.9055	235.2292
TC0300001892.mm.1	Gm9845	583.6559	552.6944	248.987	2037.059	3230.136	2714.196
TC1700000149.mm.1		62.87231	71.42672	43.20082	192.0442	281.7065	288.5911
TC0900000032.mm.1	Casp4	117.9593	90.59524	77.63354	424.0934	422.8723	470.2475
TC1400000271.mm.1	Arhgef3	205.8266	159.6741	101.2143	571.6684	818.0961	709.89
TC0300002236.mm.1	Tlr2	743.2951	502.8079	248.3919	2122.301	4880.646	2168.634
TC1100002249.mm.1	Plek	4903.123	3536.382	1702.911	13796.78	15849.86	15135.33
TC1100001942.mm.1	BC018473	39.26976	43.54885	39.17316	134.3708	177.7791	176.7783
TC1400000276.mm.1		53.67082	50.93013	36.57589	222.9736	379.7454	243.4025
TC0900003004.mm.1	Alas1	266.8549	203.2945	137.3387	745.298	961.6032	891.6802
TC0600003307.mm.1	Dusp16	120.8061	124.7411	87.10162	474.2016	624.5852	544.784
TC1800000626.mm.1	Mir6983	214.7521	201.3079	53.8163	505.2194	1542.761	953.6726
TC0700000499.mm.1		578.185	494.0424	261.2866	2099.831	2363.348	2276.693
TC0700002503.mm.1	Bcl3	80.9007	65.37894	66.9016	288.7338	332.2807	293.7526
TC1600001106.mm.1	Mefv	72.23286	74.23808	78.24474	293.8792	401.6177	321.2243
TC0600003237.mm.1	Olr1	1282.827	1266.211	401.2242	4014.516	5389.017	5666.648
TC0600000995.mm.1	Asprv1	373.4702	350.5948	108.7562	1322.966	1738.574	1591.443
TC0700002951.mm.1	Siglece	5730.751	4112.623	1869.433	14136.65	16787.04	16366.52
TC0600001530.mm.1	Clec2d	1227.929	1118.623	472.7018	4532.842	5024.976	5601.606
TC1500001498.mm.1		828.1386	769.6112	505.5889	2602.116	3651.816	3383.768
TC1500000037.mm.1	Fyb	800.9683	652.1776	356.607	2393.367	3031.105	2651.13
TC1700000934.mm.1	Treml4	161.1655	131.1231	97.06339	538.1602	523.7283	704.0037
TC1900000690.mm.1		75.48119	65.1375	74.505	299.6308	309.1635	372.1859
TC0600001770.mm.1		350.3676	370.735	195.5244	994.8544	1488.121	1424.355
TC0100000439.mm.1	Cflar	380.0822	328.1524	164.9459	1057.114	1529.584	1410.406
TC0200001928.mm.1		113.8372	86.66714	94.99969	346.7868	394.2057	385.5231
TC0200001927.mm.1	Bcl2l11	272.4955	247.4272	139.4313	784.6704	1112.923	1028.594
TC1700000214.mm.1	Fpr2; Fpr3	1254.116	1274.215	738.5472	3396.227	4835.772	5096.528
TC1500001499.mm.1		119.4167	69.87032	60.41859	248.6383	293.1464	269.9145
TC0200004093.mm.1		246.5589	188.1915	119.4207	367.14	732.5167	673.4913
TC1300002749.mm.1		62.41801	59.72511	75.7907	255.9145	222.2781	233.0279
TC0600003229.mm.1	Cd69	94.66441	95.93931	62.40875	360.2134	430.8929	335.4839
TC0100003057.mm.1	Ikbke	51.83819	52.50799	58.82616	154.6411	204.206	194.248
TC1500000578.mm.1	Gm19945	4452.21	7743.238	2781.719	14997.26	17100.92	20030.59
TC1500001495.mm.1		278.7009	176.4303	94.00003	638.8946	782.7598	513.4363
TC0900002536.mm.1		842.9477	408.8464	241.1247	1569.531	2578.018	1628.057
TC0600000266.mm.1		362.2386	246.2907	170.4779	786.5358	907.7474	1099.752
TC1700000992.mm.1	Ebi3	156.3319	142.6552	68.0518	345.1635	546.3279	556.7548
TC0100001937.mm.1	Gm15452	9439.648	16474.48	5250.833	29390.83	33776.58	43635.33
TC1900000439.mm.1	Jak2	1220.733	1019.498	506.4841	3066.851	4506.777	3908.687
TC0200003246.mm.1		119.0295	91.48304	99.38892	360.4972	458.7411	317.7433
TC0100002320.mm.1		157.1623	187.754	144.6208	449.8616	597.3099	690.1632
TC1800000625.mm.1	Csf1r	382.0212	292.2788	145.1043	854.7659	1191	995.5465
TC0600003238.mm.1		320.0097	368.438	157.1598	1240.204	1406.508	1083.962
TC0200003474.mm.1		329.6641	325.1286	175.8942	1159.119	1449.714	1016.835
TC1400000275.mm.1		51.96669	42.58802	47.91652	141.8993	205.1257	168.4525
TC1700000674.mm.1	Mir6973a	49.38726	43.23679	47.23704	137.493	175.4285	164.9363
TC1700002352.mm.1	C3	15279.07	12003.86	5363.369	33243.84	41222.14	44299.78
TC0300003013.mm.1	Dapp1	190.7904	185.7347	112.143	461.7498	637.1233	648.3563
TC1200000745.mm.1		1232.96	1379.842	854.3146	3533.019	4385.049	4163.226
TC1000002320.mm.1	Arid5b	373.5184	275.6925	146.4044	678.8401	1014.915	902.1226
TC0X00003437.mm.1	Il2rg	245.5422	226.9556	169.9002	547.3063	790.3091	753.3122
TC0200001346.mm.1	Gm18953	13011.43	17633.75	7544.229	38118.32	51538.82	41123.86
TC1100002373.mm.1	Gm20645; RP23-164N15.3	1613.438	1677.618	486.6352	4721.912	7119.229	5352.803

TC0400001384.mm.1	Gm12891	1788.74	2423.874	1158.26	5283.894	5755.789	7581.98
TC0200001371.mm.1	Acp2	112.3932	83.40883	54.44053	227.3483	304.1359	271.9722
TC1600000179.mm.1	Abcc1	97.77427	83.50668	86.43365	239.7667	334.2758	281.464
TC0700000562.mm.1	Nfkbid	191.6645	175.7659	102.7564	516.569	710.9683	589.5476
TC1200001232.mm.1		102.5594	83.48581	50.65035	243.8384	278.4624	255.5481
TC0400002079.mm.1	Nadk	7124.389	5208.579	2407.441	12644.81	17550.79	16000.22
TC1100003097.mm.1	Cxcl16	70.59137	66.30511	70.92533	208.2978	265.4818	222.9845
TC1800000673.mm.1		214.0937	199.831	68.87122	773.7957	644.1049	578.2488
TC1500001658.mm.1	St3gal1	277.4669	222.2858	140.1142	522.5228	775.4353	691.6745
TC1300002001.mm.1	Gm15440	226.1619	153.1077	162.9532	312.7957	486.051	538.9666
TC1000001478.mm.1		36.87027	32.01542	32.34714	85.45078	103.607	116.4907
TC0200003473.mm.1	Zeb2; Mir5129	250.9353	234.3319	152.5827	674.8496	869.2234	770.9621
TC1500001500.mm.1		330.2668	306.8417	176.9109	766.0522	1025.553	1255.174
TC0500000170.mm.1	Gsap	2761.047	2324.975	1050.66	6425.039	7977.175	7762.559
TC0400003680.mm.1	Smpdl3b	45.73875	43.74505	43.24398	122.877	165.3744	135.0608
TC1900000111.mm.1	Ehd1	223.7635	180.8867	143.5215	447.2744	607.7372	566.1788
TC0700000826.mm.1	Rras	133.8185	90.9332	97.92333	271.612	300.5383	321.2205
TC1500001494.mm.1		581.6547	452.8877	283.3148	1168.097	1619.756	1319.504
TC1300002002.mm.1	Sema4d	494.6293	408.1044	268.7271	1030.08	1406.817	1210.737
TC0200003792.mm.1		105.1608	98.41008	65.62763	215.9734	343.8456	310.7348
TC1400002744.mm.1	Gpr18	121.6549	107.8396	81.49229	316.1606	328.1988	260.8576
TC1300000913.mm.1		332.1894	299.6736	159.463	820.7172	971.4731	1108.529
TC0200003208.mm.1		55.54987	43.53084	39.11223	119.2941	159.9405	134.026
TC1500001492.mm.1		246.6326	217.8537	105.6331	565.9831	697.5923	735.3777
TC0300002994.mm.1	Nfkb1	659.3809	516.2567	285.439	1266.342	1757.202	1513.344
TC1800000672.mm.1	Malt1	540.4717	455.104	252.9939	1293.351	1550.179	1361.313
TC0400000590.mm.1		227.3689	288.514	131.797	451.1266	646.7639	667.1902
TC1100002374.mm.1	Rel	4088.147	4219.243	1847.516	10968.19	13362.06	12617.54
TC1100003396.mm.1	Ccl5	76.88084	75.46417	63.88011	235.2897	321.2316	221.0925
TC0600000610.mm.1		826.2527	646.0883	459.5637	1497.059	2185.48	1929.829
TC0700002696.mm.1	Zfp36	274.3768	229.0822	93.71184	663.8252	834.1382	743.3378
TC1300002748.mm.1	Zfp131	91.50877	81.423	57.47948	221.9878	262.6696	235.63
TC1300000915.mm.1		132.4385	107.0282	89.20791	287.0162	358.2854	317.3363
TC0200004089.mm.1	Gm13767	2308.615	2517.432	1608.554	5510.243	7084.167	6823.056
TC1300000560.mm.1	Ninj1	149.3937	128.403	82.70341	324.4362	412.0553	369.3441
TC0600001769.mm.1	2810474O19Rik	1267.165	1281.172	563.4484	3154.383	3781.967	3698.274
TC1400000851.mm.1	Spata13	122.0255	104.2281	92.54237	302.87	311.7505	305.33
TC0600002641.mm.1	Mxd1	23173.13	20044.03	9056.232	62013.95	68237.5	62116.77
TC1900001007.mm.1	LOC100862023	1405.031	1336.623	1002.073	3960.601	3954.171	4456.291
TC0600003308.mm.1		49.33302	43.77548	56.99401	138.0979	201.4207	146.2679
TC0200003245.mm.1	Ntng2; 6530402F18Rik	158.3751	148.4908	83.64822	369.3246	509.8958	446.2199
TC1100000363.mm.1		345.3618	319.0261	204.6789	947.2728	858.5351	973.9597
TC1200001231.mm.1	Traf3	221.7734	160.135	115.4245	380.1887	567.3484	445.3782
TC0900002535.mm.1	Gm19279	954.5739	1123.767	431.8941	2648.718	2887.804	3322.924
TC1700000932.mm.1	Trem1	2896.403	2436.266	1620.126	5971.152	6694.41	7171.901
TC1900000991.mm.1	Cdc42ep2	45.86155	40.43047	31.45227	109.98	107.0799	117.5123
TC1300000477.mm.1		80.46268	86.1049	75.44193	168.4008	222.8453	221.4192
TC1200002661.mm.1	Ighv1-82	123.1666	63.80419	75.81004	156.449	216.2863	214.7124
TC0100000536.mm.1		352.2281	369.5752	160.1548	1038.782	964.4909	991.3076
TC1300001359.mm.1		3416.086	3388.354	1352.896	7347.66	11217.11	9500.841
TC0100003300.mm.1	Ier5	107.0788	87.31908	64.29158	190.6155	300.9337	228.2783
TC1000002326.mm.1		143.7471	135.9924	139.7523	375.407	506.4469	381.9303
TC0400001047.mm.1	Pde4b	88.25571	80.71629	79.13983	216.3044	217.9316	233.5262
TC1400002295.mm.1		172.5855	155.7577	89.67063	379.4197	523.4937	435.1599
TC1500002183.mm.1	Rnd1	182.5799	135.6624	121.1973	372.9166	407.697	369.8609
TC0900002806.mm.1	Bcl2a1d	149.5562	182.8952	103.8752	392.2336	428.0463	339.6207
TC0900002534.mm.1		144.8546	181.6555	61.11578	328.6154	401.2521	397.4292
TC1100000350.mm.1	Gm12112	1301.556	950.3122	553.2533	2076.06	3116.803	2355.592
TC1900001506.mm.1	Pik3ap1	799.6324	665.4108	297.6404	1568	2180.939	1922.03
TC1500001783.mm.1	Slc39a4	71.39186	58.07219	65.14488	144.6813	200.391	173.0471
TC1700000703.mm.1	Ier3	63.79137	46.7405	38.45029	122.7012	188.2346	128.6168
TC0900002823.mm.1	Tbc1d2b	645.687	547.2731	283.5363	1080.708	1525.318	1435.778
TC1800000600.mm.1		41.05774	58.96383	35.65349	76.15956	114.1206	106.3688
TC0300000567.mm.1	Il12a	80.88589	85.52868	62.28338	186.1018	220.89	213.5883
TC0100003052.mm.1	Gm25549	92.53738	85.63167	116.137	243.8407	293.9297	200.8776
TC1000000315.mm.1	Gm26535	96.05855	96.36156	116.9768	255.2743	333.6937	242.259
TC1900000018.mm.1	Chka	70.7481	76.83854	62.27562	162.8214	200.5037	179.5687

TC0900001511.mm.1	Bcl2a1c	50.59054	52.14368	33.68162	104.3487	133.9481	130.2592
TC1700000496.mm.1	Cdkn1a	694.1071	655.1729	214.3234	1269.703	1743.894	1707.985
TC1100000362.mm.1	Lcp2	2967.357	2934.368	1662.898	7624.118	7467.128	9097.658
TC0200001424.mm.1		118.4338	113.9205	90.34896	304.0667	395.8929	214.1757
TC0200004090.mm.1	Ptprj	3328.567	3108.934	1402.128	6295.769	8345.259	8057.295
TC1100001267.mm.1	Wfdc21	6746.609	6501.614	5077.784	12627.45	18733.2	16990.07
TC1000002324.mm.1		156.9769	152.7219	52.28661	413.3296	375.3894	391.4267
TC1100000614.mm.1	Irf1	298.3597	228.9235	177.0147	483.4433	674.064	589.806
TC1400002454.mm.1	Lacc1	79.56064	94.29396	85.88235	183.8805	232.8608	219.1935
TC0900001858.mm.1	Tyk2	114.417	100.0703	86.21847	206.1179	286.265	255.8043
TC0200003367.mm.1		68.63729	58.94151	53.93671	122.198	173.0251	153.9852
TC1500001491.mm.1	Trps1; Mir1907	183.0057	154.8762	101.9733	326.0424	439.4977	373.0621
TC1100000785.mm.1	Grap	191.0018	155.3692	78.89225	326.4045	475.7513	377.6489
TC0200000923.mm.1	Tank	244.3257	229.6613	111.7919	442.2459	614.7662	579.5434
TC1300000914.mm.1		376.7863	506.7895	185.3395	895.0682	977.69	878.5428
TC1000002321.mm.1		121.6149	138.6949	87.55524	250.6691	343.2089	298.3191
TC0600001890.mm.1	Tfec	86.28027	72.52888	73.94975	131.656	179.6656	186.7144
TC0900003241.mm.1	Myd88	521.7542	407.2872	268.5113	957.3823	1212.464	994.4297
TC0500003219.mm.1	Camkk2	179.951	149.0059	97.08884	302.2679	356.3104	359.6641
TC0700000498.mm.1	Paf1	229.8618	197.9903	141.8727	399.4102	477.0901	476.5461
TC1600001071.mm.1	Ets2	72.73034	71.73758	61.04539	178.9437	204.4376	178.0872
TC1300000912.mm.1	Ell2	992.4333	718.5734	360.8999	1567.918	1783.606	1655.666
TC1800000508.mm.1		621.9439	425.2443	282.6827	1096.16	1042.38	1030.388
TC1200001490.mm.1	Adam17	653.3279	588.0262	323.8924	1314.368	1759.193	1526.786
TC0600001403.mm.1	Clec4n	121.8077	210.2945	134.0119	311.6304	376.5289	316.5737
TC0600000611.mm.1		284.5058	285.4616	121.5442	500.3921	699.7344	697.5864
TC1900000078.mm.1	Rela	2248.288	1701.272	800.3601	3331.699	4585.031	3988.925
TC1000003135.mm.1	Il23a	49.81189	61.25362	50.97123	123.8044	142.2337	104.6846
TC0900002725.mm.1		28.24718	27.34558	27.18523	73.74464	67.18747	50.08696
TC0700002705.mm.1	Nfkbib	163.9116	153.8682	154.5277	377.9323	354.8448	390.1573
TC1100001659.mm.1	Gm11625	7345.008	13886.79	6154.391	15946.56	21283.77	17408.81
TC1000002322.mm.1		44.80709	73.70602	51.60273	127.5183	156.6147	129.8695
TC0900002296.mm.1	Zc3h12c	38.86554	32.62025	33.19032	79.33953	76.5677	85.2133
TC0600002978.mm.1	Rassf4	73.15424	64.00838	52.95131	139.0766	176.2783	151.0138
TC1300002756.mm.1	Gm21188	329.8317	350.8485	185.2132	717.9429	916.7069	832.7983
TC1000000225.mm.1		424.5935	733.2396	494.4959	1225.679	1554.42	1239.306
TC0500003415.mm.1	Orai2	690.6616	499.5836	296.9531	925.2098	1376.378	1180.429
TC1000001477.mm.1		37.18413	34.47124	47.54601	89.53944	92.45841	90.17673
TC0300002802.mm.1	Vcam1	273.2212	263.1175	107.7194	572.5848	844.7037	634.5128
TC1600001556.mm.1	Parp14	108.1712	106.9146	84.67126	223.3954	311.906	253.6198
TC1400001659.mm.1	Prkcd	3351.828	2860.236	1242.631	5719.594	8420.249	7563.854
TC1600000010.mm.1		91.17445	92.45703	133.7666	252.0084	217.0031	222.1788
TC1900000776.mm.1	Acsl5	349.7696	328.045	192.8775	663.4422	953.447	809.7421
TC1400001055.mm.1		1816.315	1385.627	483.3593	3351.928	4050.576	3465.441
TC1300002698.mm.1		565.7108	550.8339	262.0846	869.467	1312.246	1336.671
TC1300000917.mm.1		147.3142	94.72785	91.08617	209.5492	325.2705	170.3846
TC0300000786.mm.1	Crtc2	277.0582	260.3382	180.5	537.1075	628.4509	632.2891
TC1300002589.mm.1		6262.504	6392.025	2281.685	14078.84	29111.62	15482.55
TC1100003253.mm.1		2008.383	1535.569	781.1625	2975.709	4388.343	3337.232
TC1100003450.mm.1	Vmp1; Mir21a	186.1701	200.8851	171.3995	407.725	435.2267	457.8045
TC0700004207.mm.1	Igsf6	133661	129864.9	56826.01	309025.4	304287.7	347540.2
TC0200004574.mm.1		226.357	162.1282	138.9286	390.6848	436.4424	343.4009
TC0200002308.mm.1	Hck	1069.177	948.6301	362.597	1798.962	3032.233	2357.38
TC1900000221.mm.1	Ms4a6c	269.5731	368.6115	281.9534	513.7864	670.34	634.2897
TC0X00001256.mm.1		965.3896	822.03	545.3475	1568.372	1834.99	1852.008
TC0700003039.mm.1	Saa3	29.95374	30.87394	38.97623	66.36449	87.04732	70.44483
TC1200000653.mm.1	Gm20235	99.5281	111.1292	73.76946	181.0705	228.3701	270.3102
TC1500001493.mm.1		314.1083	365.5023	113.5974	586.9159	876.286	787.1417
TC0X00000074.mm.1	Pim2	39.04786	36.15281	37.62061	85.0602	87.15827	86.16058
TC0400001325.mm.1	Slc2a1	239.4076	223.3951	123.558	423.0059	548.9927	521.8256
TC1000002997.mm.1		82.3209	82.95139	74.7117	188.7138	187.2639	154.3474
TC0900000518.mm.1		1129.29	1148.12	597.0596	2109.886	2802.253	2610.095
TC0800000115.mm.1	F10	131.221	105.0489	84.83939	228.2413	280.7924	238.8628
TC0500003256.mm.1	Rilpl2	230.0271	187.2484	126.2233	339.9837	474.2508	416.0643
TC1900001343.mm.1		103.2994	116.2213	75.58604	201.08	225.0196	282.4922
TC0600000609.mm.1	Snx10	736.5468	595.8937	386.2102	1187.664	1412.256	1292.893

TC1800000834.mm.1	Pstpip2	681.8439	499.1809	260.7607	971.4872	1064.982	1053.16
TC0400000529.mm.1	Nr4a3	44.05305	54.41943	44.58572	91.006	117.5396	98.39037
TC1100003449.mm.1		150.0818	97.06098	83.92007	202.2652	223.3914	234.0572
TC0600001446.mm.1	Chd4; Mir7045	556.7471	435.3199	229.0108	767.5587	1118.676	932.3975
TC0500003417.mm.1	Sh2b2	98.97578	87.46336	62.68258	157.2962	189.2178	197.7009
TC0100001633.mm.1		97.58924	132.7973	88.62476	165.0688	225.2956	255.0863
TC1700001319.mm.1	Foxn2	549.5951	456.5156	239.2434	926.0866	1114.323	1015.069
TC1200002440.mm.1	Gpr132	79.8746	91.34167	89.92309	200.6274	177.2512	202.2336
TC0200000837.mm.1		143.5038	113.9889	64.51718	242.5776	303.7544	240.4485
TC1300000476.mm.1	Hivep1	273.5495	236.6332	177.6358	399.6276	500.8726	515.8896
TC0300000911.mm.1		45.24813	39.303	33.93959	85.98414	87.90936	76.69032
TC1000000933.mm.1	Txnrd1	131.8972	112.9154	80.39064	221.7316	291.1678	238.3556
TC0600000265.mm.1	Klhdc10	183.6773	163.7045	118.9918	341.4346	372.9403	351.6949
TC0200001795.mm.1		191.1336	151.848	97.21078	270.8865	346.9304	323.8628
TC0200001345.mm.1	Gm13768	290.2836	302.7477	213.0827	614.5939	672.4819	650.809
TC0200000252.mm.1	Gm13312	37.89075	40.95805	34.43702	75.75436	82.66573	89.93765
TC1600001919.mm.1	Samsn1	235.4427	250.6815	166.8583	514.0103	529.3645	542.2618
TSUnmapped00000047.mm.1	Cd83	55.52198	48.45886	38.81018	106.1681	145.4805	99.42759
TC0700003794.mm.1		179.2715	155.5653	123.6192	277.5691	394.2914	331.4401
TC1600001005.mm.1	Ifnar1	1122.219	901.967	403.3599	1731.097	2245.347	1978.737
TC0900002803.mm.1		565.6805	465.7521	222.3759	889.1892	1032.767	1027.607
TC0800001010.mm.1	2010110E17Rik; RP23-279M5.1	64.38534	64.54057	68.13979	140.1358	145.3329	128.5551
TC0500000891.mm.1		87.42316	90.40186	67.04745	216.4814	194.8033	161.9008
TC1700000948.mm.1	Mocs1	419.4316	360.2444	221.6274	637.7119	779.7201	864.4811
TC1300000268.mm.1	Hist1h2aa	183.1114	258.7796	119.3757	375.0536	383.5533	402.5653
TC0700002491.mm.1	Relb	99.20477	92.25105	96.41703	192.2574	233.7417	204.4846
TC0500003463.mm.1	Pilra	7710.13	6988.066	3843.028	15787.84	15721.95	23024.4
TC0400000589.mm.1	Tmem38b	504.0509	420.6177	274.4982	761.2908	1049.64	855.8905
TC1300000600.mm.1		30.45586	38.79984	38.55043	78.51787	83.19765	82.48175
TC1000003214.mm.1	Lilrb4a	158496.7	125943.2	67163.28	218867	260661.1	254570.6
TC0600001911.mm.1	Gm20186	3077.843	4890.408	2748.464	5301.398	6431.009	6830.854
TC0600000236.mm.1	Irf5	118.3849	112.0039	81.39819	190.655	249.5494	240.6953
TC0900001446.mm.1		287.4518	293.8617	227.4482	612.9431	622.1063	495.5699
TC1900001339.mm.1		42.56406	45.4947	39.71361	91.66652	105.3073	71.24538
TC0600002194.mm.1	Rbpsuh-rs3	1564.422	1322.12	707.4236	2360.722	3095.877	2866.605
TC0300000210.mm.1	Skil	3889.902	3071.215	1389.673	5799.523	6393.708	6415.901
TC1800000013.mm.1	4833419F23Rik	52.59832	48.73289	28.20279	72.16094	104.0729	107.7974
TC1300000157.mm.1	Aoah	348.6586	319.6666	166.1798	617.3909	691.4957	703.7948
TC1200001491.mm.1		74.48388	67.35396	57.26734	106.0273	139.0711	150.6877
TC0700003833.mm.1		56.5932	64.53021	40.07784	114.2869	108.3222	125.6146
TC0400000581.mm.1		212.0412	162.1467	105.7848	258.1234	322.386	340.2713
TC0200000838.mm.1		261.7252	228.7402	129.2836	415.8174	599.5481	496.2295
TC0100002326.mm.1		140.747	164.5577	108.2445	307.0294	204.471	283.1141
TC1600000936.mm.1	Mir155	59.57175	81.21371	116.4835	169.7803	172.0921	192.0936
TC1500001370.mm.1	Rnf19a; Mir8097	274.4851	251.6879	170.1269	531.0412	704.5568	431.0105
TC1100000475.mm.1	Havcr2	66.00098	55.28841	46.39923	101.6929	115.3148	119.7915
TC0500003685.mm.1	N4bp2l1	367.5538	299.3356	150.9001	557.5326	690.5192	610.4861
TC1800000341.mm.1		91.46172	186.5449	145.0386	284.7803	233.9378	299.0831
TC1600000009.mm.1	Zfp263	77.82809	68.08708	64.19505	133.0329	179.0339	142.243
TC1300000603.mm.1		60.50914	58.79343	64.13857	110.0687	141.9958	126.8216
TC1000002647.mm.1	Spic	55.79373	64.84574	48.53208	115.0903	119.441	99.28481
TC1000000316.mm.1		32.54927	37.58053	42.7084	54.41603	80.41453	97.18091
TC0600003484.mm.1	Gm10388	108.1795	61.7878	73.39481	129.8605	165.7909	157.1698
TC0100001397.mm.1	Stx6	120.0389	103.6451	79.07285	176.39	211.3457	206.7777
TC1500001496.mm.1		116.6071	119.9857	64.03316	235.4974	380.1907	249.138
TC0500000738.mm.1	Rest	216.217	189.533	113.1361	399.8947	447.1447	389.0676
TC0100001222.mm.1	Chil1	7946.602	6926.71	4014.481	14408.13	14776.13	17822.27
TC1300000479.mm.1		97.77744	118.7128	99.06699	179.655	217.8368	202.737
TC0200005314.mm.1	Ctsz	737.3073	637.0622	267.1078	1099.006	1862.819	1359.154
TC0X00003182.mm.1	Gm15151	1045.892	1602.953	710.7211	2148.849	2010.462	2738.792
TC0800001037.mm.1	Nod2	60.21405	58.65696	67.21937	108.7557	122.6432	120.1895
TC0700002128.mm.1	Tarm1	1103.515	934.48	614.9467	1640.043	1805.376	2187.481
TC0200001794.mm.1	Snap23	4343.894	3520.216	2206.456	6360.817	6905.241	7305.37
TC0200000839.mm.1		176.0462	202.6332	74.86214	377.0983	385.5509	460.4726
TC0700004005.mm.1		470.7663	630.9718	419.2752	842.5098	988.6533	1070.629
TC1900001123.mm.1	AW112010	57.41093	52.8539	49.33101	83.16254	109.1678	121.7624

TC1700001027.mm.1	Tnfsf9	17.36746	16.90017	17.93394	30.6014	48.63181	34.35268
TC1100000364.mm.1		281.2482	330.7673	153.9454	492.7711	669.191	572.2328
TC1900001164.mm.1		63.45415	55.40794	42.67807	93.39147	108.2973	124.1502
TC0700003834.mm.1	Atg16l2	123.789	107.1157	101.1598	190.0393	238.0036	219.8968
TC0600001258.mm.1	Irak2	229.4808	210.5253	128.7833	386.3121	505.4325	435.044
TC0500003465.mm.1	Pilrb2	1005.388	850.3644	434.7424	1573.142	1779.545	1904.725
TC1200001149.mm.1	AF357426	36.83585	24.89387	34.14934	17.45836	17.38122	24.98785
TC0800001847.mm.1	Gm24423	91.41019	97.99602	154.7711	72.91724	48.16838	46.77374
TC0700003160.mm.1	Gm24799	255.1723	569.6785	337.7754	195.3538	160.6786	175.447
TC0700003142.mm.1	Gm25121	149.6135	169.6414	180.9441	81.25226	68.87497	107.6001
TC0600001933.mm.1	Gm24217	35.73639	55.33845	65.25947	21.80404	30.87128	27.21089
TC1400001834.mm.1	Gm17204; Gm17124; [illegible]	239.8238	383.742	357.0094	183.398	168.7002	197.1166
TC0Y00000332.mm.1	Gm20827	213.9227	370.7884	257.932	131.636	132.127	163.5923
TC0700003317.mm.1	Gm26097	82.24298	155.9369	82.42365	41.39214	35.12929	44.46331
TC1400001396.mm.1	Gm26746	614.4985	1085.606	841.9219	401.4065	367.1082	482.4447
TC0400000980.mm.1		65.41364	58.16713	63.0997	39.03428	31.53512	30.25565
TC1300002586.mm.1		56.73442	76.95835	80.0371	38.26304	32.06704	40.08617
TC0Y00000116.mm.1	Gm21898	176.2221	222.7225	252.8971	88.21783	105.6018	133.6684
TC0500002130.mm.1	Speer4b	237.0257	385.7451	348.0023	153.906	171.9208	193.0053
TC1400001829.mm.1	Gm2951	279.2045	573.5402	341.9853	161.4928	199.9609	171.2135
TC1400001801.mm.1	Gm7970	368.1505	477.7731	554.2691	266.0141	226.3796	229.4661
TC1200001872.mm.1		9075.8	14331.21	14224.31	6864.931	6948.668	7245.264
TC0Y00000454.mm.1	Gm21757	102.1972	133.3291	140.1969	65.86449	60.00211	87.13992
TC0Y00000428.mm.1	Gm21848	102.1972	133.3291	140.1969	65.86449	60.00211	87.13992
TC0Y00000369.mm.1	Gm21840	102.1972	133.3291	140.1969	65.86449	60.00211	87.13992
TC0400000516.mm.1	Gm12424	45789.04	77774.79	55968.76	24467.92	31723.62	27971.91
TC0Y00000501.mm.1	Gm21919	220.91	282.9814	345.6825	113.9528	131.5324	175.045
TC0700003153.mm.1	Gm26498	172.0169	238.9659	181.3656	79.12317	91.05655	85.70801
TC0700003144.mm.1	Gm26230	172.0169	238.9659	181.3656	79.12317	91.05655	85.70801
TC1400001350.mm.1	Gm25272	52.64009	69.2623	96.49125	43.11185	34.00449	33.42414
TC0Y00000459.mm.1	Gm20865; Gm20800	242.1078	302.1089	294.926	153.8602	145.1649	130.2783
TC0Y00000432.mm.1	Gm20865; Gm20933	242.1078	302.1089	294.926	153.8602	145.1649	130.2783
TC0Y00000373.mm.1	Gm20865; Gm21285	242.1078	302.1089	294.926	153.8602	145.1649	130.2783
TC0200001682.mm.1		35848.88	44729.8	38694.05	18766.5	18871.61	20938.19
TC0200002719.mm.1	Gm14288; Gm14440	1217.559	2070.938	1835.438	877.2023	877.8271	876.1372
TC4_JH584293_random00000011.mm.1		422.3845	825.083	689.1006	319.1584	307.8386	330.5002
TC0600002655.mm.1		60134.33	43033.46	77358.14	27840.26	17721.32	28658.17
TC1400001812.mm.1	Gm6401	280.8124	482.66	408.4563	189.9407	185.3288	212.183
TC1100004056.mm.1	Gm11709	1106.719	1088.364	993.4178	783.7057	530.1622	516.7022
TC0700003274.mm.1	Gm22131	175.1048	408.7975	213.3038	94.01146	101.5427	115.7315
TC0700003097.mm.1	LOC100861735	39.76029	31.7236	34.63446	17.19927	16.13755	26.47363
TC0X00000286.mm.1	Gm2837	147.0092	213.156	227.9322	105.353	103.087	83.52222
TC0700003185.mm.1	Gm25874	221.8607	449.9044	267.0338	133.3653	174.0496	126.8297
TC0Y00000070.mm.1	Gm20809	1738.532	4196.937	2204.918	1086.141	880.4509	1086.469
TC0700003182.mm.1	Gm22111	301.7411	375.4229	326.4358	150.2589	243.7674	124.7047
TC0700003181.mm.1	Gm24566	301.7411	375.4229	326.4358	150.2589	243.7674	124.7047
TC0700003179.mm.1	Gm22393	301.7411	375.4229	326.4358	150.2589	243.7674	124.7047
TC0700003178.mm.1	Gm23687	301.7411	375.4229	326.4358	150.2589	243.7674	124.7047
TC0700003174.mm.1	Gm26389	301.7411	375.4229	326.4358	150.2589	243.7674	124.7047
TC0400003988.mm.1	Gm15951	159.0237	156.7921	145.9234	75.54091	61.10999	88.3052
TC1400000058.mm.1	Gm3208	471.3835	675.0754	704.7366	377.0692	327.4977	316.9843
TC0700003154.mm.1	Gm23660	224.1721	531.0756	376.389	168.6396	122.0017	183.9234
TC1100001897.mm.1		3588.461	4723.12	5025.776	2225.227	2056.276	2554.678
TC0700003304.mm.1	Gm22776	237.1364	541.0977	344.7553	176.0442	163.6672	159.7137
TC0700003297.mm.1	Gm24618	639.5504	2061.346	774.3704	322.1998	335.0699	403.8615
TC0700003296.mm.1	Gm22863	639.5504	2061.346	774.3704	322.1998	335.0699	403.8615
TC0700003294.mm.1	Gm26332	639.5504	2061.346	774.3704	322.1998	335.0699	403.8615
TC0700003293.mm.1	Gm23953	639.5504	2061.346	774.3704	322.1998	335.0699	403.8615
TC0700003278.mm.1	Gm24264	1064.429	2903.408	1097.926	431.1909	546.0592	510.2694
TC1400001813.mm.1	Gm3543	329.826	597.4777	604.5319	278.2839	239.9479	277.1661
TC1400001795.mm.1	Gm6482	500.9206	1087.626	631.7961	299.1113	314.0302	413.3533
TC0Y00000461.mm.1	Gm21855	220.7457	286.0315	293.507	109.3666	129.6362	183.7386
TC0Y00000063.mm.1	Gm21907	220.7457	286.0315	293.507	109.3666	129.6362	183.7386
TC1100002722.mm.1	Gm11187; RP23-388J21.1	4439.831	8394.95	7141.533	3007.192	3577.583	3187.451
TC0Y00000487.mm.1	Gm20917	3663.768	8555.89	3914.492	1756.432	1737.21	2046.858
TC0X00000276.mm.1	Gm21637; Spin2-ps1	180.2537	250.7333	243.6567	120.7128	97.82583	113.9163

TC1400001860.mm.1	Gm17654	345.3872	697.4398	547.551	242.977	233.2332	308.6271
TC1000000963.mm.1		94.72497	165.601	112.8532	52.89359	46.29477	82.6739
TC1200000190.mm.1	Gm26140	4060.836	9825.998	7326.44	2781.531	3138.715	3530.748
TC0700003210.mm.1	Gm24495	99.01238	179.685	129.6256	64.39887	60.01046	50.32901
TC1400001803.mm.1	Gm3072	287.8568	606.6315	383.1203	177.5255	179.461	245.275
TC0700003284.mm.1	Gm22584	1971.785	4916.335	1712.984	681.156	823.2225	1003.732
TC1400001817.mm.1	Gm7951	610.3316	1333.258	817.9674	381.5664	381.1714	514.71
TC1000001264.mm.1		473477.3	846528.1	670014.3	276504.3	289160.3	304548.9
TC0Y00000565.mm.1	Gm20854; Gm20862; Gm20863	5139.413	14217.49	6938.107	3271.26	3262.483	3426.476
TC0Y00000120.mm.1	Gm21878	179.1474	253.4211	211.6665	105.2595	81.60502	95.81139
TC0X00001561.mm.1		473477.3	846528.1	670014.3	276504.3	289160.3	304548.9
TC0400002484.mm.1		1682.967	3033.52	2229.813	903.8524	982.9312	1222.129
TC0200004488.mm.1	Gm14001	59.91349	96.42965	99.84493	44.09784	41.91255	54.38992
TC0Y00000429.mm.1	Gm20936	2632.667	6763.984	2945.387	1245.911	1649.253	1220.393
TC0Y00000370.mm.1	Gm21268	2632.667	6763.984	2945.387	1245.911	1649.253	1220.393
TC0Y00000556.mm.1	Ssty1; Gm20910	735.4288	1573.503	1302.017	567.0819	481.0801	630.2723
TC0Y00000555.mm.1	Ssty1	735.4288	1573.503	1302.017	567.0819	481.0801	630.2723
TC0Y00000068.mm.1	Gm20809	533.2701	915.0347	726.3175	314.1621	312.9957	237.272
TC1400001367.mm.1	Gm2866	374.2868	604.5182	654.5219	327.4358	234.9178	273.8872
TC1300002220.mm.1		1020.759	2403.136	1767.427	731.4841	614.7784	901.0099
TC0Y00000280.mm.1	LOC100862366	127.803	260.2954	155.5302	70.792	69.88707	105.4973
TC0Y00000272.mm.1	LOC100042565	127.803	260.2954	155.5302	70.792	69.88707	105.4973
TC0600000199.mm.1		11918.28	30059.12	15906.55	7433.28	7267.646	9516.511
TC0Y00000163.mm.1	Gm21843	388.7314	843.8085	605.4545	248.6902	180.8708	264.9692
TC0700003159.mm.1	Gm26466	42.78174	41.02973	58.24575	17.5498	18.93762	33.10654
TC0800002159.mm.1		27509.47	31568.24	17207.9	12595.41	11846.54	11205.19
TC0400002197.mm.1		23665.07	35384.72	40056.27	15232.77	15072.29	16970.1
TC1400001810.mm.1	Gm7980	353.1598	521.6255	539.7463	252.6185	225.5729	230.4493
TC1400001362.mm.1	Gm26817	434.818	539.6175	540.161	277.0448	178.7189	238.7508
TC0Y00000061.mm.1	Gm20917; Gm21469	4579.809	8192.872	4036.987	1837.731	2196.948	1677.727
TC1500001918.mm.1		756.2939	798.1132	967.535	356.3005	327.4899	654.3008
TC1400001407.mm.1	Gm3476	464.0754	1007.032	833.2966	351.1838	348.4215	388.4362
TC0X00001744.mm.1		370.7041	930.7663	662.7987	268.7775	208.9955	271.8139
TC0700003146.mm.1	Gm25314	202.1974	285.2821	280.961	129.8638	99.23704	121.8844
TC1400001824.mm.1	Gm17026	177.6606	356.1915	292.0474	121.2979	118.5389	147.9848
TC0Y00000446.mm.1	Gm21808	3938.894	10949.04	5261.75	2395.615	2168.781	2370.411
TC0Y00000057.mm.1	Gm20747; Gm21683; Gm20909	4163.941	9269.385	5831.376	2608.235	2189.169	2858.939
TC1600001949.mm.1		319.5888	238.4197	422.4912	110.6031	170.9157	134.5344
TC1900001143.mm.1		27613.21	68744.4	37422.32	9101.977	20083.46	16840.92
TC1800001393.mm.1		27613.21	68744.4	37422.32	9101.977	20083.46	16840.92
TC1600000722.mm.1		27613.21	68744.4	37422.32	9101.977	20083.46	16840.92
TC1200002489.mm.1		27613.21	68744.4	37422.32	9101.977	20083.46	16840.92
TC1200001838.mm.1		27613.21	68744.4	37422.32	9101.977	20083.46	16840.92
TC1200001486.mm.1		27613.21	68744.4	37422.32	9101.977	20083.46	16840.92
TC1200000168.mm.1		27613.21	68744.4	37422.32	9101.977	20083.46	16840.92
TC1000002465.mm.1		27613.21	68744.4	37422.32	9101.977	20083.46	16840.92
TC1000001319.mm.1		27613.21	68744.4	37422.32	9101.977	20083.46	16840.92
TC0Y00000474.mm.1	Gm21875	665.7927	1729.605	923.3646	467.4016	375.5374	413.9959
TC0X00001200.mm.1		27613.21	68744.4	37422.32	9101.977	20083.46	16840.92
TC0X00001094.mm.1		27613.21	68744.4	37422.32	9101.977	20083.46	16840.92
TC0X00000800.mm.1		27613.21	68744.4	37422.32	9101.977	20083.46	16840.92
TC0800000456.mm.1		27613.21	68744.4	37422.32	9101.977	20083.46	16840.92
TC0200001615.mm.1		27613.21	68744.4	37422.32	9101.977	20083.46	16840.92
TC0200001569.mm.1		27613.21	68744.4	37422.32	9101.977	20083.46	16840.92
TC0100001327.mm.1		27613.21	68744.4	37422.32	9101.977	20083.46	16840.92
TC0100001326.mm.1		27613.21	68744.4	37422.32	9101.977	20083.46	16840.92
TC0100001325.mm.1		27613.21	68744.4	37422.32	9101.977	20083.46	16840.92
TC0900000155.mm.1	Olfr58	39.87895	72.21152	88.61456	27.01517	29.37209	32.67593
TC0700003260.mm.1	Gm22253	1057.712	2375.875	1782.63	893.1149	730.23	557.5503
TC0200005327.mm.1	Gm16354	579.3898	966.2222	793.54	326.9189	281.5605	372.9122
TC1300001454.mm.1		17394.96	42153.82	26577.87	6865.789	12736	11173.58
TC1400001387.mm.1	Gm3140	429.2962	608.5147	571.8914	349.3754	250.2526	240.9824
TC0700003148.mm.1	Gm22996	335.1968	736.4572	490.4206	210.2647	145.3254	201.5449
TC0700003145.mm.1	Gm23922	335.1968	736.4572	490.4206	210.2647	145.3254	201.5449
TC0700003143.mm.1	Gm24657	335.1968	736.4572	490.4206	210.2647	145.3254	201.5449
TC0Y00000263.mm.1	Gm20737	414.2048	894.6163	537.1256	228.3803	184.2373	310.4002
TC1300001682.mm.1		391922.7	747531.1	608801.5	228655.8	238541.7	269756.4

TC0Y00000257.mm.1	Gm21721	632.4437	1691.895	1087.432	413.642	447.7105	436.6667
TC0900000023.mm.1		391922.7	747531.1	608801.5	228655.8	238541.7	269756.4
TC0100000346.mm.1		391922.7	747531.1	608801.5	228655.8	238541.7	269756.4
TC1600000909.mm.1		3280.344	5609.339	5516.056	1414.782	2369.837	2177.476
TC0Y00000520.mm.1	Gm21879	3433.804	9967.548	4759.067	2049.129	1947.109	2307.873
TC0Y00000026.mm.1	Gm20821	328.6949	550.8823	375.7608	156.6168	138.6272	161.9556
TC0700003140.mm.1		725.1287	1805.812	1289.529	509.2305	405.4919	593.1545
TC0600001311.mm.1		3280.344	5609.339	5516.056	1414.782	2369.837	2177.476
TC0700003164.mm.1	Gm25229	546.9291	741.6097	882.509	190.7204	324.1852	290.0804
TC0Y00000124.mm.1	Gm21735	4547.344	14146.82	7086.115	3060.35	2578.014	3234.311
TC0800002100.mm.1	Gm23128	57.59687	84.68162	82.51458	56.1189	34.46481	33.08623
TC0700003219.mm.1	Gm25647	320.063	677.2928	467.1736	180.6874	235.0907	190.2189
TC0700003218.mm.1	Gm25823	320.063	677.2928	467.1736	180.6874	235.0907	190.2189
TC0700003324.mm.1	Gm25615	783.9979	2339.122	1185.058	343.7736	485.3601	520.5777
TC0Y00000531.mm.1	Gm21862	242.8584	367.5836	405.1128	153.5456	147.7982	212.9869
TC0Y00000527.mm.1	Gm21892	242.8584	367.5836	405.1128	153.5456	147.7982	212.9869
TC0Y00000416.mm.1	Gm21877	3131.989	8841.041	5007.782	2009.165	1498.2	2086.819
TC0Y00000355.mm.1	Gm21923	3131.989	8841.041	5007.782	2009.165	1498.2	2086.819
TC0100003247.mm.1		256.4165	252.9913	168.7666	99.90102	118.646	102.5221
TC0Y00000529.mm.1	Gm21881	8140.329	25335.08	12951.59	5359.966	4839.485	6033.537
TC0Y00000525.mm.1	Gm21785	8140.329	25335.08	12951.59	5359.966	4839.485	6033.537
TC1800001019.mm.1		8230.267	13363.2	16459.77	4227.507	6227.926	4969.414
TC1800000926.mm.1		8230.267	13363.2	16459.77	4227.507	6227.926	4969.414
TC1700002716.mm.1		8230.267	13363.2	16459.77	4227.507	6227.926	4969.414
TC1700001176.mm.1		8230.267	13363.2	16459.77	4227.507	6227.926	4969.414
TC1700000889.mm.1		8230.267	13363.2	16459.77	4227.507	6227.926	4969.414
TC1600000908.mm.1		8230.267	13363.2	16459.77	4227.507	6227.926	4969.414
TC1600000815.mm.1		8230.267	13363.2	16459.77	4227.507	6227.926	4969.414
TC1500001604.mm.1		8230.267	13363.2	16459.77	4227.507	6227.926	4969.414
TC1500000476.mm.1		8230.267	13363.2	16459.77	4227.507	6227.926	4969.414
TC1500000343.mm.1		8230.267	13363.2	16459.77	4227.507	6227.926	4969.414
TC1400001079.mm.1		8230.267	13363.2	16459.77	4227.507	6227.926	4969.414
TC1300002612.mm.1		8230.267	13363.2	16459.77	4227.507	6227.926	4969.414
TC1300002385.mm.1		8230.267	13363.2	16459.77	4227.507	6227.926	4969.414
TC1200000401.mm.1		8230.267	13363.2	16459.77	4227.507	6227.926	4969.414
TC1000002362.mm.1		8230.267	13363.2	16459.77	4227.507	6227.926	4969.414
TC0X00003262.mm.1		8230.267	13363.2	16459.77	4227.507	6227.926	4969.414
TC0X00002870.mm.1		8230.267	13363.2	16459.77	4227.507	6227.926	4969.414
TC0X00002594.mm.1		8230.267	13363.2	16459.77	4227.507	6227.926	4969.414
TC0X00002104.mm.1		8230.267	13363.2	16459.77	4227.507	6227.926	4969.414
TC0900001177.mm.1		8230.267	13363.2	16459.77	4227.507	6227.926	4969.414
TC0900000039.mm.1		8230.267	13363.2	16459.77	4227.507	6227.926	4969.414
TC0900000012.mm.1		8230.267	13363.2	16459.77	4227.507	6227.926	4969.414
TC0800002239.mm.1		8230.267	13363.2	16459.77	4227.507	6227.926	4969.414
TC0800001136.mm.1		8230.267	13363.2	16459.77	4227.507	6227.926	4969.414
TC0800000500.mm.1		8230.267	13363.2	16459.77	4227.507	6227.926	4969.414
TC0700004091.mm.1		8230.267	13363.2	16459.77	4227.507	6227.926	4969.414
TC0600002387.mm.1		8230.267	13363.2	16459.77	4227.507	6227.926	4969.414
TC0600000209.mm.1		8230.267	13363.2	16459.77	4227.507	6227.926	4969.414
TC0500002431.mm.1		8230.267	13363.2	16459.77	4227.507	6227.926	4969.414
TC0500002430.mm.1		8230.267	13363.2	16459.77	4227.507	6227.926	4969.414
TC0300001918.mm.1		8230.267	13363.2	16459.77	4227.507	6227.926	4969.414
TC0300000093.mm.1		8230.267	13363.2	16459.77	4227.507	6227.926	4969.414
TC0200002991.mm.1		8230.267	13363.2	16459.77	4227.507	6227.926	4969.414
TC0100001930.mm.1		8230.267	13363.2	16459.77	4227.507	6227.926	4969.414
TC0100001122.mm.1		8230.267	13363.2	16459.77	4227.507	6227.926	4969.414
TC0400003312.mm.1		31530.88	42976.41	70559.75	22230.89	13526.71	17783.05
TC1900001634.mm.1		6549.322	13130.37	10432.69	2657.432	4840.864	4051.353
TC1800001570.mm.1		6549.322	13130.37	10432.69	2657.432	4840.864	4051.353
TC1800000768.mm.1		6549.322	13130.37	10432.69	2657.432	4840.864	4051.353
TC1500000481.mm.1		6549.322	13130.37	10432.69	2657.432	4840.864	4051.353
TC1400002705.mm.1		6549.322	13130.37	10432.69	2657.432	4840.864	4051.353
TC0Y00000534.mm.1	Gm20860	6257.779	20310.72	9670.26	4054.134	3622.428	4487.914
TC0Y00000470.mm.1	Gm20813	6257.779	20310.72	9670.26	4054.134	3622.428	4487.914
TC0X00003344.mm.1		6549.322	13130.37	10432.69	2657.432	4840.864	4051.353
TC0X00000379.mm.1		6549.322	13130.37	10432.69	2657.432	4840.864	4051.353

TC0900002567.mm.1		6549.322	13130.37	10432.69	2657.432	4840.864	4051.353
TC0600003306.mm.1		6549.322	13130.37	10432.69	2657.432	4840.864	4051.353
TC0600002537.mm.1		6549.322	13130.37	10432.69	2657.432	4840.864	4051.353
TC0600001731.mm.1		6549.322	13130.37	10432.69	2657.432	4840.864	4051.353
TC0300000439.mm.1		6549.322	13130.37	10432.69	2657.432	4840.864	4051.353
TC1400001128.mm.1		6549.322	13130.37	10432.69	2657.432	4840.864	4051.353
TC0Y00000129.mm.1	Gm20793	384.2479	848.3303	608.3567	219.3022	165.2452	361.4789
TC0Y00000076.mm.1	Gm20809; Gm21443	1260.76	2831.749	1763.833	779.9294	705.8635	708.8706
TC1500001674.mm.1		2278.428	3573.392	4293.771	1338.356	1393.474	1207.234
TC1300000419.mm.1		30898.33	70460.66	60121.21	24154.23	17576.5	24314.33
TC0700003194.mm.1	Gm25175	198.7248	502.9722	405.348	154.1055	126.1781	155.0867
TC0Y00000181.mm.1	Gm21806	2247.255	5227.881	3659.343	1332.721	1114.038	1371.274
TC1600001649.mm.1		15899.48	30591.33	24101.48	6546.867	11260.06	9051.636
TC1500001159.mm.1		15899.48	30591.33	24101.48	6546.867	11260.06	9051.636
TC1400001168.mm.1		15899.48	30591.33	24101.48	6546.867	11260.06	9051.636
TC1300001262.mm.1		15899.48	30591.33	24101.48	6546.867	11260.06	9051.636
TC1100002198.mm.1		15899.48	30591.33	24101.48	6546.867	11260.06	9051.636
TC1000001265.mm.1		15899.48	30591.33	24101.48	6546.867	11260.06	9051.636
TC0800000165.mm.1		15899.48	30591.33	24101.48	6546.867	11260.06	9051.636
TC0800000071.mm.1		15899.48	30591.33	24101.48	6546.867	11260.06	9051.636
TC0300001517.mm.1		15899.48	30591.33	24101.48	6546.867	11260.06	9051.636
TC0100001321.mm.1		15899.48	30591.33	24101.48	6546.867	11260.06	9051.636
TC0Y00000085.mm.1	Gm21804	3769.159	11529.1	6285.356	2408.443	2273.482	2656.395
TC0400002440.mm.1		13863.04	21211.38	17875.63	6386.649	9408.755	6825.284
TC1200001839.mm.1		3915.489	11599.55	7455.015	2679.622	2442.728	2908.181
TC0400000538.mm.1	Gm12437; RP23-141C15.4	26238.01	61221.09	46647.99	16411.33	16785.09	18981.07
TC1000001909.mm.1	Mir680-3	30247.08	54062.25	54062.25	18107.95	20495.22	21731.63
TC0Y00000503.mm.1	Gm21728	5056.009	16408.6	8277.899	3233.432	3171.778	3419.547
TC0Y00000258.mm.1	Gm21812	2519.032	8143.731	4644.399	1686.893	1717.282	1708.125
TC0Y00000447.mm.1	Gm20866	562.1326	1120.513	991.9703	413.4689	337.7723	391.3069
TC0Y00000418.mm.1	Gm21530	562.1326	1120.513	991.9703	413.4689	337.7723	391.3069
TC0Y00000356.mm.1	Gm20880	562.1326	1120.513	991.9703	413.4689	337.7723	391.3069
TC0900000022.mm.1		71932.38	156711.5	145853.8	55931.85	47537.85	54738.3
TC0Y00000050.mm.1	Gm20851	441.2186	1023.677	663.504	235.01	194.5246	407.4976
TC0Y00000035.mm.1	Gm20773	441.2186	1023.677	663.504	235.01	194.5246	407.4976
TC0700003200.mm.1	Gm22632	1346.479	3873.746	2744.719	929.9705	882.9315	1116.365
TC0700003198.mm.1	Gm24926	1346.479	3873.746	2744.719	929.9705	882.9315	1116.365
TC1300000992.mm.1		500.3693	931.3417	658.5742	249.0527	233.9665	468.1979
TC0Y00000121.mm.1	Gm20922	2345.779	6337.859	3194.549	1239.483	1086.191	1353.359
TC0Y00000021.mm.1	Gm21778	1070.825	2740.061	1819.117	629.0598	642.9436	834.5452
TC1100003399.mm.1	Ccl6	9755.775	7351.629	4108.691	2531.63	2460.579	2913.946
TC0Y00000254.mm.1	Gm20825	4740.812	15572.75	8347.758	3027.294	2606.592	3488.854
TC0Y00000074.mm.1	Gm20809; Gm21427	2413.236	5933.619	3605.968	1300.939	930.5042	1323.23
TC0100002724.mm.1		684.7147	1699.389	1397.382	455.3703	495.2303	558.3953
TC0Y00000477.mm.1	Gm21163	5036.292	17161.03	8170.017	3018.426	3087.324	3697.628
TC0Y00000186.mm.1	Gm20818	5036.292	17161.03	8170.017	3018.426	3087.324	3697.628
TC0200004195.mm.1	Gm13805; RP23-392O16.1	18684.87	53210.87	28552.56	8092.29	10682.09	11751.53
TC0Y00000064.mm.1	Gm20925	5343.237	15189.47	7958.419	2606.32	3232.983	2961.531
TC0100002881.mm.1		7473.898	13459.7	13211.96	3599.314	5407.816	4695.026
TC0900002568.mm.1		524269.3	995531.9	957387.8	333153	321696.7	385498.6
TC0Y00000491.mm.1	Gm20806; Gm21248	1358.304	3957.906	2521.941	791.7298	834.746	1097.955
TC0500000573.mm.1		6509.191	16964.69	13211.95	3591.924	4147.747	5782.06
TC0100000997.mm.1		6509.191	16964.69	13211.95	3591.924	4147.747	5782.06
TC0700003306.mm.1	Gm25210	1516.41	4281.917	2578.548	622.7239	829.5461	886.7146
TC0700003303.mm.1	Gm25471	1516.41	4281.917	2578.548	622.7239	829.5461	886.7146
TC0700003298.mm.1	Gm22046	1516.41	4281.917	2578.548	622.7239	829.5461	886.7146
TC0700003295.mm.1	Gm26032	1516.41	4281.917	2578.548	622.7239	829.5461	886.7146
TC0Y00000500.mm.1	Gm20747; Gm20801	4910.603	10745.86	6899.419	2363.593	1926.292	2634.976
TC0700003137.mm.1		674.0721	1330.055	1251.501	418.883	417.0051	435.6909
TC0X00000580.mm.1	Gm14658; RP23-226L1.2	2987.943	6629.319	6363.681	2069.851	1995.632	2233.082
TC0700003309.mm.1	Gm25074	983.3718	2601.412	1604.066	397.9489	521.5338	637.6693
TC0Y00000374.mm.1	Gm20747	5102.134	11721.64	7558.701	2458.789	1912.276	2947.896
TC0Y00000460.mm.1	Gm20747; Gm21683	4908.601	10918.54	7340.509	2262.834	1963.91	2813.062
TC0800000144.mm.1		2657.851	5549.917	5859.212	1757.941	1777.843	1821.988
TC0700003191.mm.1		935.9452	2302.26	1907.476	596.4722	613.0869	739.254
TC1100004055.mm.1		210.4275	208.457	134.7106	57.2732	63.00338	81.37759
TC0700003311.mm.1	Gm22631	1005.558	2612.773	1664.51	412.9576	494.4453	629.9611

TC0Y00000530.mm.1	Gm21435	5965.495	17673.42	8474.593	2690.845	2386.327	3459.879
TC0Y00000072.mm.1	Gm20809; Gm21412	3005.862	9057.125	5298.609	1636.503	1557.915	2077.581
TC0Y00000486.mm.1	Gm20919	6324.972	17213.98	9273.565	2759.35	2949.71	3001.309
TC0Y00000062.mm.1	Gm21661	6324.972	17213.98	9273.565	2759.35	2949.71	3001.309
TC0700003272.mm.1	Gm23862	1051.505	3146.27	1868.308	433.0175	539.2056	714.7366
TC0700003340.mm.1	Gm26094	1555.824	4255.654	2464.743	577.9382	756.9677	828.6555
TC0700003339.mm.1	Gm23696	1555.824	4255.654	2464.743	577.9382	756.9677	828.6555
TC0700003336.mm.1	Gm24742	1555.824	4255.654	2464.743	577.9382	756.9677	828.6555
TC0700003335.mm.1	Gm23359	1555.824	4255.654	2464.743	577.9382	756.9677	828.6555
TC0700003334.mm.1	Gm24658	1555.824	4255.654	2464.743	577.9382	756.9677	828.6555
TC0700003333.mm.1	Gm26136	1555.824	4255.654	2464.743	577.9382	756.9677	828.6555
TC0700003331.mm.1	Gm23524	1555.824	4255.654	2464.743	577.9382	756.9677	828.6555
TC0700003330.mm.1	Gm26223	1555.824	4255.654	2464.743	577.9382	756.9677	828.6555
TC0700003328.mm.1	Gm23549	1555.824	4255.654	2464.743	577.9382	756.9677	828.6555
TC0700003326.mm.1	Gm23724	1555.824	4255.654	2464.743	577.9382	756.9677	828.6555
TC0700003325.mm.1	Gm22110	1555.824	4255.654	2464.743	577.9382	756.9677	828.6555
TC0700003323.mm.1	Gm23357	1555.824	4255.654	2464.743	577.9382	756.9677	828.6555
TC0700003322.mm.1	Gm26433	1555.824	4255.654	2464.743	577.9382	756.9677	828.6555
TC0700003321.mm.1	Gm22812	1555.824	4255.654	2464.743	577.9382	756.9677	828.6555
TC0700003319.mm.1	Gm26201	1555.824	4255.654	2464.743	577.9382	756.9677	828.6555
TC0700003316.mm.1	Gm25597	1555.824	4255.654	2464.743	577.9382	756.9677	828.6555
TC0700003314.mm.1	Gm26246	1555.824	4255.654	2464.743	577.9382	756.9677	828.6555
TC0700003307.mm.1	Gm22128	1555.824	4255.654	2464.743	577.9382	756.9677	828.6555
TC0700003300.mm.1	Gm25350	1555.824	4255.654	2464.743	577.9382	756.9677	828.6555
TC0700003337.mm.1	Snord116l1	1507.809	4014.526	2339.851	547.6733	712.6697	881.269
TC0700003329.mm.1	Snord116l2	1507.809	4014.526	2339.851	547.6733	712.6697	881.269
TC0700003299.mm.1	Snord116l1; Snord116l2; Snord116	1507.809	4014.526	2339.851	547.6733	712.6697	881.269
TC0700003289.mm.1	Snord116l1; Snord116l2; Snord116	1507.809	4014.526	2339.851	547.6733	712.6697	881.269
TC0700003285.mm.1	Snord116l1; Snord116l2; Snord116	1507.809	4014.526	2339.851	547.6733	712.6697	881.269
TC0700003283.mm.1	Snord116l1; Snord116l2; Snord116	1507.809	4014.526	2339.851	547.6733	712.6697	881.269
TC0700003280.mm.1	Snord116l1; Snord116l2; Snord116	1507.809	4014.526	2339.851	547.6733	712.6697	881.269
TC0700003279.mm.1	Snord116l1; Snord116l2; Snord116	1507.809	4014.526	2339.851	547.6733	712.6697	881.269
TC0700003277.mm.1	Snord116l1; Snord116l2; Snord116	1507.809	4014.526	2339.851	547.6733	712.6697	881.269
TC0700003275.mm.1	Snord116l1; Snord116l2; Snord116	1507.809	4014.526	2339.851	547.6733	712.6697	881.269
TC0700003273.mm.1	Snord116l1; Snord116l2; Snord116	1507.809	4014.526	2339.851	547.6733	712.6697	881.269
TC1700001735.mm.1		118.2251	78.08311	109.6759	65.54298	32.24376	32.65303
TC0700003327.mm.1	Gm22188	1289.212	3185.635	2088.917	425.3022	594.7532	701.2462
TC1200001680.mm.1		86850.13	235783.5	221840.2	64436.77	52205.36	70128.23
TC0500002444.mm.1		285828.5	583829.5	526656.3	142148.4	149619.3	185273.1
TC0500002445.mm.1		6182.273	18352.28	11571.29	2941.196	2254.792	3729.938
TC1700002364.mm.1		88278.78	292399.9	232674.8	60931.08	46809.26	67847.71
TC0100000178.mm.1		88278.78	292399.9	232674.8	60931.08	46809.26	67847.71
TC0Y00000421.mm.1	Gm21654	6707.624	14375.85	8391.077	2078.61	1941.361	2831.577
TC0Y00000362.mm.1	Gm20884	6707.624	14375.85	8391.077	2078.61	1941.361	2831.577
TC1_GL456221_random00000023.mm.1		11911.88	35157.37	33901.93	5350.521	7128.252	8285.421
TC1_GL456221_random00000006.mm.1		11911.88	35157.37	33901.93	5350.521	7128.252	8285.421
TC1_GL456212_random00000012.mm.1		11911.88	35157.37	33901.93	5350.521	7128.252	8285.421
TC1_GL456212_random00000002.mm.1		11911.88	35157.37	33901.93	5350.521	7128.252	8285.421
TC1_GL456211_random00000017.mm.1		11911.88	35157.37	33901.93	5350.521	7128.252	8285.421
TC1_GL456211_random00000012.mm.1		11911.88	35157.37	33901.93	5350.521	7128.252	8285.421
TC0500003459.mm.1		11911.88	35157.37	33901.93	5350.521	7128.252	8285.421
TC0100002706.mm.1		11911.88	35157.37	33901.93	5350.521	7128.252	8285.421
TC0100000753.mm.1		11911.88	35157.37	33901.93	5350.521	7128.252	8285.421
TC0100000748.mm.1		11911.88	35157.37	33901.93	5350.521	7128.252	8285.421
TC0100000739.mm.1		11911.88	35157.37	33901.93	5350.521	7128.252	8285.421
TC0100000730.mm.1		11911.88	35157.37	33901.93	5350.521	7128.252	8285.421
TC1200001840.mm.1		361.9865	164.4737	458.3344	60.81231	84.48653	46.46943
