# Supplementary material for: Activated Neutrophils Secrete Chitinase-Like 1 and Attenuate Liver Inflammation by Inhibiting Pro-Inflammatory Macrophage Responses
Source: Front Immunol. 2022 Apr 21;13:824385. doi: 10.3389/fimmu.2022.824385 (PMC9069964; doi:10.3389/fimmu.2022.824385)
Supplement: Supplementary file 2 [file Table_2.pdf]

Supplementary table 2. GO terms of genes in S1P-activated neutrophils

<table>
<tr><th>#Term</th><th>ID</th><th>P-Value</th><th>Corrected P-Value</th><th>input_gene</th></tr>
<tr><td>inflammatory response</td><td>GO:0006954</td><td>1.06745E-30</td><td>1.2857E-27</td><td>Rela,19697:up|Cd14,12475:up|Ccrl2,54199:up|C3,12266:up|Irak2,108960:up|Cxcl2,20310:up|Adam17,11491:up|Pik3ap1,83490:up|Tlr2,24088:up|Il1b,16176:up|Tnfrsf1b,21938:up|Nlrp3,216799:up|Nfe2l2,18024:up|Il23a,83430:up|Nfkb2,18034:up|Ccl5,20304:up|Cxcl10,15945:up|Jak2,16452:up|Casp4,12363:up|Il1rn,16181:up|Relb,19698:up|Mapkapk2,17164:up|Smpdl3b,100340:up|Zfp36,22695:up|Icam1,15894:up|Chil1,12654:up|Ccl6,20305:down|Siglece,83382:up|Aoah,27052:up|Ccl3,20302:up|Saa3,20210:up|Fas,14102:up|Olr1,108078:up|Acod1,16365:up|Nfkb1,18033:up|Tnip1,57783:up|Hck,15162:up|Il1a,16175:up|Mefv,54483:up|Tnfaip3,21929:up|Nfkbiz,80859:up|Tnf,21926:up|Snap23,20619:up|Myd88,17874:up|Ptgs2,19225:up|Nfkbid,243910:up|Csf1r,12978:up|Zc3h12a,230738:up|Havcr2,171285:up</td></tr>
<tr><td>cytokine production</td><td>GO:0001816</td><td>2.46688E-28</td><td>2.3587E-25</td><td>Rela,19697:up|Malt1,240354:up|Ltb,16994:up|Cd14,12475:up|C3,12266:up|Adam17,11491:up|Clec4n,56620:up|Tlr2,24088:up|Il1b,16176:up|Ifnar1,15975:up|Nlrp3,216799:up|Il23a,83430:up|Cd83,12522:up|Ccl5,20304:up|Jak2,16452:up|Cd274,60533:up|Casp4,12363:up|Relb,19698:up|Prkcd,18753:up|Clec5a,23845:up|Mapkapk2,17164:up|Clec4e,56619:up|Icosl,50723:up|Zfp36,22695:up|Traf3,22031:up|Chil1,12654:up|Il12a,16159:up|Ccl3,20302:up|Irf1,16362:up|Lcp2,16822:up|Bcl3,12051:up|Nr4a3,18124:up|Acod1,16365:up|Nfkb1,18033:up|Il1a,16175:up|Mefv,54483:up|Tnfaip3,21929:up|Tnf,21926:up|Trem1,58217:up|Myd88,17874:up|Ptgs2,19225:up|Csf1r,12978:up|Gpr18,110168:up|Irak3,73914:up|Zc3h12a,230738:up|Havcr2,171285:up|Tnfsf9,21950:up</td></tr>
<tr><td>regulation of cytokine production</td><td>GO:0001817</td><td>4.55082E-26</td><td>2.1926E-23</td><td>Rela,19697:up|Malt1,240354:up|Ltb,16994:up|Cd14,12475:up|C3,12266:up|Adam17,11491:up|Clec4n,56620:up|Tlr2,24088:up|Il1b,16176:up|Ifnar1,15975:up|Nlrp3,216799:up|Il23a,83430:up|Cd83,12522:up|Ccl5,20304:up|Jak2,16452:up|Cd274,60533:up|Casp4,12363:up|Relb,19698:up|Clec5a,23845:up|Mapkapk2,17164:up|Clec4e,56619:up|Icosl,50723:up|Zfp36,22695:up|Traf3,22031:up|Il12a,16159:up|Ccl3,20302:up|Irf1,16362:up|Bcl3,12051:up|Nr4a3,18124:up|Acod1,16365:up|Nfkb1,18033:up|Il1a,16175:up|Mefv,54483:up|Tnfaip3,21929:up|Tnf,21926:up|Myd88,17874:up|Ptgs2,19225:up|Gpr18,110168:up|Csf1r,12978:up|Irak3,73914:up|Zc3h12a,230738:up|Havcr2,171285:up|Tnfsf9,21950:up</td></tr>
<tr><td>regulation of immune system process</td><td>GO:0002682</td><td>7.34992E-25</td><td>2.951E-22</td><td>Clec2d,93694:up|Samsn1,67742:up|Rela,19697:up|Hcar2,80885:up|Malt1,240354:up|Cd14,12475:up|C3,12266:up|Sh2b2,23921:up|Irak2,108960:up|Cxcl2,20310:up|Birc3,11796:up|Adam17,11491:up|Ptprj,19271:up|Pik3ap1,83490:up|Tlr2,24088:up|Il1b,16176:up|Nlrp3,216799:up|Nfkbia,18035:up|Nfe2l2,18024:up|Il23a,83430:up|Cd83,12522:up|Ccl5,20304:up|Cxcl10,15945:up|Cd274,60533:up|Vcam1,22329:up|Mapkapk2,17164:up|Smpdl3b,100340:up|Clec4e,56619:up|Icosl,50723:up|Zfp36,22695:up|Il2rg,16186:up|Icam1,15894:up|Traf3,22031:up|Il12a,16159:up|Ccl3,20302:up|Irf1,16362:up|Lcp2,16822:up|Fas,14102:up|Nr4a3,18124:up|Acod1,16365:up|Tnip1,57783:up|Il1a,16175:up|Tnfaip3,21929:up|Clec4d,17474:up|Tnf,21926:up|Paf1,54624:up|Trem1,58217:up|Myd88,17874:up|Nfkbid,243910:up|Csf1r,12978:up|Cdkn1a,12575:up|Gpr18,110168:up|Irak3,73914:up|Zc3h12a,230738:up|Havcr2,171285:up|Ifnlr1,242700:up|Tnfsf9,21950:up</td></tr>
<tr><td>response to cytokine</td><td>GO:0034097</td><td>5.84001E-23</td><td>1.6551E-20</td><td>Rela,19697:up|Cd14,12475:up|Sh2b2,23921:up|Irak2,108960:up|Cxcl2,20310:up|Il1b,16176:up|Ifnar1,15975:up|Nfkbia,18035:up|Nfe2l2,18024:up|Wfdc21,66107:up|Tank,21353:up|Nfkb2,18034:up|Ccl5,20304:up|Cxcl10,15945:up|Jak2,16452:up|Cd274,60533:up|Il1rn,16181:up|Relb,19698:up|Mapkapk2,17164:up|Zfp36,22695:up|Traf3,22031:up|Ccl6,20305:down|Cxcl16,66102:up|Ccl3,20302:up|Saa3,20210:up|Irf1,16362:up|Acod1,16365:up|Nfkb1,18033:up|Il1a,16175:up|Mefv,54483:up|Tnf,21926:up|Irf5,27056:up|Myd88,17874:up|Ptgs2,19225:up|Ikbke,56489:up|Csf1r,12978:up|Irak3,73914:up|Zc3h12a,230738:up|Ifnlr1,242700:up</td></tr>
</table>

<table>
<tr><td>cell communication</td><td>GO:0007154</td><td>2.42186E-19</td><td>4.1673E-17</td><td>Rnd1,223881:up|Rela,19697:up|Hcar2,80885:up|Cd14,12475:up|Ccrl2,54199:up|Rest,19712:up|C3,12266:up|Rassf4,213391:up|Ptprj,19271:up|Gpr132,56696:up|Pik3ap1,83490:up|Ifnar1,15975:up|Nfe2l2,18024:up|Il23a,83430:up|Cd83,12522:up|Ccl5,20304:up|Fpr1,14293:up|Arhgef3,71704:up|Cd274,60533:up|Skil,20482:up|Il1rn,16181:up|Pilrb2,545812:up|Arid5b,71371:up|Vcam1,22329:up|Slc39a4,72027:up|Mapkapk2,17164:up|Map3k8,26410:up|Sqstm1,18412:up|F10,14058:up|Gpr84,80910:up|Clec4e,56619:up|Icosl,50723:up|Zfp36,22695:up|Traf3,22031:up|Sod2,20656:up|Ccl6,20305:down|Adgre1,13733:up|Saa3,20210:up|Bcl3,12051:up|Cflar,12633:up|Fas,14102:up|Pilra,231805:up|Nr4a3,18124:up|Bcl2l11,12125:up|Nfkb1,18033:up|Acod1,16365:up|Hck,15162:up|Tnfaip3,21929:up|Clec4d,17474:up|Paf1,54624:up|Pim2,18715:up|Trem1,58217:up|Myd88,17874:up|Ikbke,56489:up|Gpr18,110168:up|Irak3,73914:up|Spata13,219140:up|Zc3h12a,230738:up|Havcr2,171285:up|Atg16l2,73683:up|Plek,56193:up|Fpr3,14294:up|Malt1,240354:up|Sh2b2,23921:up|Irak2,108960:up|Adam17,11491:up|Birc3,11796:up|Cxcl2,20310:up|Clec4n,56620:up|Tlr2,24088:up|Nr4a1,15370:up|Il1b,16176:up|Tnfrsf1b,21938:up|Nlrp3,216799:up|Nfkbia,18035:up|Zeb2,24136:up|Fpr2,14289:up|Tank,21353:up|Nfkb2,18034:up|Fyb,23880:up|Gadd45b,17873:up|Cxcl10,15945:up|Jak2,16452:up|Casp4,12363:up|Relb,19698:up|Prkcd,18753:up|Slc2a1,20525:up|Smpdl3b,100340:up|Icam1,15894:up|Chil1,12654:up|Il12a,16159:up|Nfkbib,18036:up|Ccl3,20302:up|Bcl2a1a,12044:up|Irf1,16362:up|Lcp2,16822:up|Cdc42ep2,104252:up|Sema4d,20354:up|Rras,20130:up|Tnip1,57783:up|Il1a,16175:up|Tnf,21926:up|Snap23,20619:up|Irf5,27056:up|Ptgs2,19225:up|Nfkbid,243910:up|Csf1r,12978:up|Cdkn1a,12575:up|Tbc1d2b,67016:up|Ifnlr1,242700:up|Tnfsf9,21950:up</td></tr>
<tr><td>cell activation</td><td>GO:0001775</td><td>3.90618E-17</td><td>5.0865E-15</td><td>Samsn1,67742:up|Malt1,240354:up|Adam17,11491:up|Ptprj,19271:up|Ebi3,50498:up|Tlr2,24088:up|Il1b,16176:up|Ifnar1,15975:up|Nlrp3,216799:up|Il23a,83430:up|Nfkb2,18034:up|Cd83,12522:up|Ccl5,20304:up|Cxcl10,15945:up|Jak2,16452:up|Cd274,60533:up|Relb,19698:up|Vcam1,22329:up|Prkcd,18753:up|Clec4e,56619:up|Icosl,50723:up|Icam1,15894:up|Il2rg,16186:up|Il12a,16159:up|Slc7a11,26570:up|Irf1,16362:up|Lcp2,16822:up|Bcl3,12051:up|Fas,14102:up|Nr4a3,18124:up|Clec4d,17474:up|Tnfaip3,21929:up|Tnf,21926:up|Snap23,20619:up|Myd88,17874:up|Nfkbid,243910:up|Gpr18,110168:up|Cdkn1a,12575:up|Havcr2,171285:up|Plek,56193:up|Tnfsf9,21950:up</td></tr>
<tr><td>cytokine secretion</td><td>GO:0050663</td><td>3.07478E-14</td><td>2.2446E-12</td><td>Chil1,12654:up|Cd14,12475:up|Ccl3,20302:up|Lcp2,16822:up|Clec4n,56620:up|Tlr2,24088:up|Il1b,16176:up|Ifnar1,15975:up|Nlrp3,216799:up|Il1a,16175:up|Tnf,21926:up|Cd274,60533:up|Trem1,58217:up|Casp4,12363:up|Csf1r,12978:up|Clec5a,23845:up|Zc3h12a,230738:up|Havcr2,171285:up|Clec4e,56619:up</td></tr>
<tr><td>regulation of cell communication</td><td>GO:0010646</td><td>1.88181E-09</td><td>5.4949E-08</td><td>Rela,19697:up|Hcar2,80885:up|Malt1,240354:up|Rest,19712:up|C3,12266:up|Sh2b2,23921:up|Irak2,108960:up|Birc3,11796:up|Adam17,11491:up|Ptprj,19271:up|Pik3ap1,83490:up|Clec4n,56620:up|Tlr2,24088:up|Il1b,16176:up|Tnfrsf1b,21938:up|Nlrp3,216799:up|Nfkbia,18035:up|Nfe2l2,18024:up|Zeb2,24136:up|Il23a,83430:up|Tank,21353:up|Ccl5,20304:up|Gadd45b,17873:up|Cxcl10,15945:up|Jak2,16452:up|Arhgef3,71704:up|Skil,20482:up|Il1rn,16181:up|Prkcd,18753:up|Sqstm1,18412:up|Smpdl3b,100340:up|F10,14058:up|Icam1,15894:up|Chil1,12654:up|Sod2,20656:up|Il12a,16159:up|Nfkbib,18036:up|Ccl6,20305:down|Ccl3,20302:up|Irf1,16362:up|Cflar,12633:up|Sema4d,20354:up|Fas,14102:up|Rras,20130:up|Bcl2l11,12125:up|Acod1,16365:up|Nfkb1,18033:up|Tnip1,57783:up|Il1a,16175:up|Tnfaip3,21929:up|Tnf,21926:up|Pim2,18715:up|Myd88,17874:up|Ptgs2,19225:up|Nfkbid,243910:up|Csf1r,12978:up|Irak3,73914:up|Spata13,219140:up|Zc3h12a,230738:up|Tbc1d2b,67016:up|Havcr2,171285:up|Plek,56193:up</td></tr>
<tr><td>extracellular space</td><td>GO:0005615</td><td>0.000250029</td><td>0.00178996</td><td>Chil1,12654:up|Il12a,16159:up|Ccl6,20305:down|Ltb,16994:up|Cd14,12475:up|Cxcl16,66102:up|C3,12266:up|Ccl3,20302:up|Cxcl2,20310:up|Saa3,20210:up|Ebi3,50498:up|Sema4d,20354:up|Il1b,16176:up|Fas,14102:up|Il1a,16175:up|Il23a,83430:up|Wfdc21,66107:up|Tnf,21926:up|Ccl5,20304:up|Cxcl10,15945:up|Il1rn,16181:up|Ctsz,64138:up|Vcam1,22329:up|Slc2a1,20525:up|Smpdl3b,100340:up|Tnfsf9,21950:up|Icam1,15894:up</td></tr>
</table>
